# Oxidation of PKGIα mediates an endogenous adaptation to pulmonary hypertension

**DOI:** 10.1073/pnas.1904064116

**Published:** 2019-06-11

**Authors:** Olena Rudyk, Alice Rowan, Oleksandra Prysyazhna, Susanne Krasemann, Kristin Hartmann, Min Zhang, Ajay M. Shah, Clemens Ruppert, Astrid Weiss, Ralph T. Schermuly, Tomoaki Ida, Takaaki Akaike, Lan Zhao, Philip Eaton

**Affiliations:** ^a^School of Cardiovascular Medicine & Sciences, British Heart Foundation Centre of Excellence, King’s College London, London, United Kingdom;; ^b^Institute of Neuropathology, University Medical Centre Hamburg-Eppendorf, Hamburg, Germany;; ^c^Universities of Giessen & Marburg Lung Center Giessen Biobank, Justus-Liebig-University Giessen, Giessen, Germany;; ^d^Excellence Cluster Cardio-Pulmonary System, Justus-Liebig-University Giessen, Giessen, Germany;; ^e^Department of Environmental Medicine and Molecular Toxicology, Tohoku University Graduate School of Medicine, Sendai, Japan;; ^f^Faculty of Medicine, Department of Medicine, Imperial College London, London, United Kingdom

**Keywords:** pulmonary hypertension, protein kinase G, hypoxia, redox, oxidative stress

## Abstract

Chronic hypoxia causes pulmonary hypertension (PH), vascular remodeling, right ventricular (RV) hypertrophy, and cardiac failure. Protein kinase G Iα (PKGIα) is susceptible to oxidation, forming an interprotein disulfide homodimer associated with kinase targeting involved in vasodilation. Here we report increased disulfide PKGIα in pulmonary arteries from mice with hypoxic PH or lungs from patients with pulmonary arterial hypertension. This oxidation is likely caused by oxidants derived from NADPH oxidase-4, superoxide dismutase 3, and cystathionine γ-lyase, enzymes that were concomitantly increased in these samples. Indeed, products that may arise from these enzymes, including hydrogen peroxide, glutathione disulfide, and protein-bound persulfides, were increased in the plasma of hypoxic mice. Furthermore, low-molecular-weight hydropersulfides, which can serve as “superreductants” were attenuated in hypoxic tissues, consistent with systemic oxidative stress and the oxidation of PKGIα observed. Inhibiting cystathionine γ-lyase resulted in decreased hypoxia-induced disulfide PKGIα and more severe PH phenotype in wild-type mice, but not in Cys42Ser PKGIα knock-in (KI) mice that are resistant to oxidation. In addition, KI mice also developed potentiated PH during hypoxia alone. Thus, oxidation of PKGIα is an adaptive mechanism that limits PH, a concept further supported by polysulfide treatment abrogating hypoxia-induced RV hypertrophy in wild-type, but not in the KI, mice. Unbiased transcriptomic analysis of hypoxic lungs before structural remodeling identified up-regulation of endothelial-to-mesenchymal transition pathways in the KI compared with wild-type mice. Thus, disulfide PKGIα is an intrinsic adaptive mechanism that attenuates PH progression not only by promoting vasodilation but also by limiting maladaptive growth and fibrosis signaling.

## Significance

This study demonstrates that oxidation of protein kinase G Iα (PKGIα) to its disulfide-activated state occurs in pulmonary arteries during chronic hypoxia, and that this is a protective event that limits progression of pulmonary hypertension by at least two mechanisms. Firstly, it induces pulmonary vasodilation that counters and offsets maladaptive vasoconstriction during chronic hypoxia, and, secondly, disulfide PKGIα is protective by preventing maladaptive growth and fibrosis signaling. Consistent with oxidation of PKGIα being protective, administration of polysulfides to mice during hypoxia, which increased the abundance of the disulfide form of the kinase, was therapeutic and limited disease progression.

Hypoxic pulmonary vasoconstriction is a physiological response that enhances blood oxygenation during localized alveolar hypoxia. When larger territories become hypoxic, as occurs with high-altitude living or with lung disease, this promotes sustained pulmonary hypertension (PH) and vascular remodeling involving right ventricular (RV) hypertrophy, cardiac failure, and premature death (1–3). Prolonged hypoxia is associated with production of oxidants (3, 4), which historically have been considered pathogenic. However, oxidants can participate in regulatory and adaptive redox signaling by reversible modification of proteins (5–7). PKG is a serine/threonine protein kinase, which phosphorylates biologically important targets, including those that regulate smooth muscle relaxation, platelet function, and cell growth and division. PKGIα is susceptible to oxidation, forming an interprotein disulfide homodimer associated with kinase targeting and activation resulting in vasodilation and cardiac diastolic relaxation (8–10). During acute hypoxia, pulmonary cells become proreducing, which may explain the conversion of disulfide PKGIα to its reduced form under such conditions (11) and so its potential contribution to hypoxic pulmonary vasoconstriction. In contrast, chronic hypoxia, a time when production of reactive oxygen species (ROS) is elevated (4, 12–15), is paradoxically associated with increased PKGI expression (16), arguably serving an adaptive mechanism to limit PH. Consistent with up-regulation of the kinase being adaptive, PKGI knock-out mice develop spontaneous PH even during normoxia (17). In addition, PKGIα overexpression reduces migration of pulmonary arterial smooth muscle cells (PASMCs) subjected to hypoxia in vitro (18), suggesting a potential therapeutic value for PKGIα in hypoxia-associated pulmonary arterial remodeling.

Posttranslational regulation of PKG has recently emerged as a topic of interest in PH. Decreased PKG activity due to tyrosine nitration has been reported (19), with this nitrative stress-mediated PKG dysfunction being associated with poorer outcomes in caveolin 1-deficient mice during PH (19). A role for disulfide PKGIα in the acute vascular responses to hypoxia has been suggested (11); however, the occurrence and role of this oxidative modification during sustained low levels of oxygen remain to be elucidated. Although oxidants have been proposed as mediators of the adverse vascular remodeling that accompanies PH (20–22), of course this mirrors a generic paradigm proposed for most diseases. This has led to antioxidant supplements being advocated as a panacea, as they have for PH (13, 23), but, in general, they have failed in large-scale clinical trials and often have proven harmful when administered to humans with various diseases (24, 25). This may be because antioxidants prevent intrinsic cellular responses (25, 26), for example by neutralizing ROS species that may otherwise react with redox sensor proteins, such as PKGIα, to initiate adaptive signaling.

The role of redox-regulated PKGIα in controlling the tone of systemic vessels has been widely reported (8–10), whereas the importance of these events in the pulmonary circulation is less well defined. In the present study, the redox state of pulmonary PKGIα and its potential role in the pathogenesis of hypoxia-induced PH was investigated. The novelty of this study is that disulfide PKGIα accumulates during chronic hypoxia and that this serves an endogenous, adaptive redox mechanism by promoting vasodilation that limits PH and the associated adverse pulmonary arterial and right heart remodeling that otherwise ensues. Furthermore, disulfide PKGIα accumulation during chronic hypoxia was associated, likely causatively, with a loss of low-molecular-weight hydropersulfides that can serve as “superreductants” that otherwise maintain the kinase in the reduced state. Pharmacological agents that induce disulfide PKGIα have therapeutic potential in PH, another original finding of this work.

## Results and Discussion

### Disulfide PKGIα Level Is Elevated in Pulmonary Tissues from Hypoxic Mice and Lungs from Pulmonary Hypertensive Patients.

**Fig. 1.**
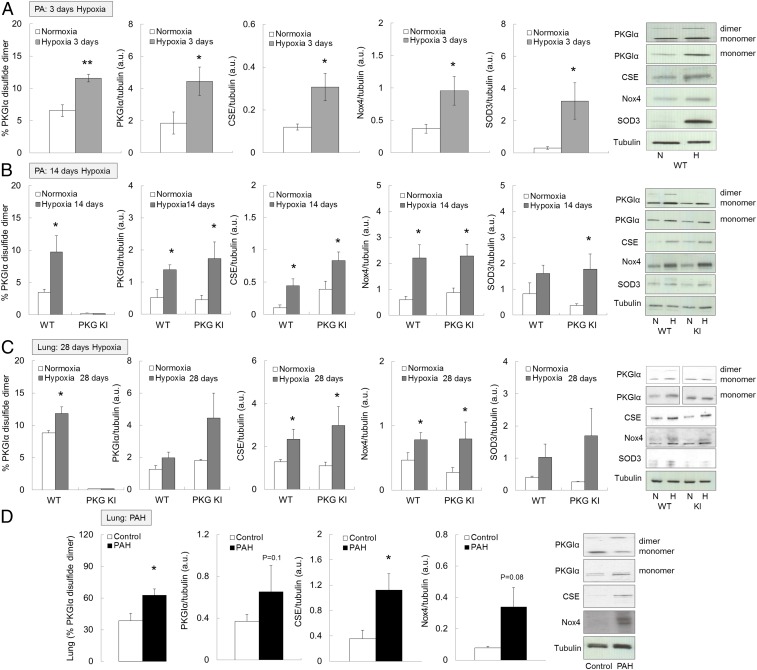
Disulfide PKGIα is increased in pulmonary arteries and lungs of mice subjected to chronic hypoxia and in lungs of PAH patients. (*A*) Disulfide PKGIα, total PKGI, CSE, Nox4, and SOD3 protein expression in vessels of WT mice subjected to either normoxia or chronic hypoxia for 3 d. (*B*) Disulfide PKGIα, total PKGI, CSE, Nox4, and SOD3 protein expression in vessels of WT or Cys42Ser PKGIα KI mice subjected to either normoxia or chronic hypoxia for 14 d. (*C*) Disulfide PKGIα, total PKGI, CSE, Nox4, and SOD3 protein expression in lungs of WT or Cys42Ser PKGIα KI mice subjected to either normoxia or chronic hypoxia for 28 d. (*D*) Disulfide PKGIα, total PKGI, CSE, and Nox4 protein expression in lungs of PAH patients. *P < 0.05, **P < 0.01 versus normoxia or respective WT; *n* = 6 to 8 per group; monomer, PKGIα monomer; dimer, PKGIα dimer; Nox4, NADPH oxidase 4; SOD, extracellular SOD; WT, WT mice; PKG KI, “redox-dead” Cys42Ser PKGIα KI mice. In some cases, the aspect ratio of the original immunoblots was altered to enable a concise multipanel figure with a consistent presentation style; the original uncropped representative images of these immunoblots are also available in *SI Appendix*, Figs. S8 and S9.

Mouse models of hypoxic PH reproduce the pulmonary vessel constriction and muscularization (27, 28), observed in humans in Group 3 of the World Health Organization PH classification system (3, 29). For this reason, we established and validated this PH model in C57BL/6 mice by subjecting them to chronic hypoxia (10% O_2_) for 28 d (*SI Appendix*, Fig. S1 *A*–*D*). Subsequently, wild-type (WT) alone, or WT together with “redox-dead” Cys42Ser PKGIα knock-in (KI) mice that cannot form PKGIα disulfide dimer (8, 10) were compared in their responses to 3, 14, or 28 d of hypoxia; 3 or 14 d of hypoxia increased the amount of total, as well as disulfide, PKGIα in WT pulmonary vessels compared with those maintained in normoxic room air (Fig. 1 *A* and *B*). Similarly, elevated disulfide PKGIα levels, as well as a trend toward increased total PKGIα, were evident in whole lungs of mice subjected to hypoxia for 28 d (Fig. 1*C*), or those from human pulmonary arterial hypertension (PAH) patients (Fig. 1*D*). This elevation in total PKGI expression was as observed by others (16), and was considered an adaptive process. However, it was unclear whether the increase in disulfide PKGIα contributes to pulmonary adaptation to hypoxia. PKGIα oxidation was not altered in the RV and was slightly reduced in the left ventricle and septum of hypoxic WT mice (*SI Appendix*, Fig. S2*A*). As anticipated, disulfide PKGIα was not evident in tissues from the KI mice regardless of the experimental intervention (Fig. 1 *B* and *C* and *SI Appendix*, Fig. S2*A*), although total PKGI was up-regulated in pulmonary vessels (Fig. 1 *A* and *B*) and lungs (Fig. 1*C*) of the KI mice in response to hypoxia, as occurred in WTs.

Next, we went on to investigate the molecular basis for pulmonary PKGIα oxidation during chronic hypoxia. Our attention turned to the ROS that might mediate oxidation of PKGIα, and their potential enzymatic sources. Previous studies showed that hydrogen peroxide (H_2_O_2_) (8, 30), persulfides (9), or nitric oxide-related species (10) induced oxidation of PKGIα. In addition, expression of the H_2_O_2_-producing superoxide dismutase (SOD) enzyme was altered in hypoxic PH (12, 31). The H_2_O_2_-generating enzyme nonphagocytic NADPH oxidase-4 (Nox4), that can also increase cystathionine γ-lyase (CSE) (32), was up-regulated in pulmonary vascular cells and pulmonary vessels during chronic hypoxia in vitro (33) and in vivo (20). For these reasons, expression of SOD, Nox4, and CSE enzymes was compared in normoxic or hypoxic tissues. Hypoxia increased Nox4, SOD3, and CSE protein expression comparably in WT or KI pulmonary arteries (Fig. 1 *A* and *B*) and lungs (Fig. 1*C*). SOD1 or SOD2 expression was not significantly altered in the lungs of mice subjected to hypoxia (*SI Appendix*, Fig. S2*B*), although the latter was previously observed to decrease in PH lungs (34, 35). To ensure accurate measurement of Nox4 protein expression, the custom-made Nox4 antibodies were validated by using Nox4-knockout lung tissue (*SI Appendix*, Fig. S2*C*).

Up-regulation of oxidant-producing proteins may reflect an adaptive role for oxidants whereby they lower pulmonary blood pressure by disulfide PKGIα-dependent vasodilation. Indeed, increased SOD3 expression is consistent with an established role for this enzyme in protecting the lung from hypoxia-induced PH (36–38). Consistent with such a protective role for the dismutase, SOD3 depletion from smooth muscle cells potentiated the severity of phenotypic responses to PH in mice (39), whereas its overexpression in lung attenuated PH-induced arterial remodeling and pressure (37). Thus, the increased SOD3 observed herein was likely an adaptive response that limits dysfunction during PH, with the rational likelihood that the increased Nox4 that also generates H_2_O_2_ is likewise beneficial. In line with this, Fawn-Hooded rats that produce less H_2_O_2_ develop spontaneous PH, compared with Sprague Dawley controls. However, treatment of Fawn-Hooded rats with an SOD2 mimetic, which is anticipated to enhance H_2_O_2_ derived from superoxide, abrogated the PH phenotype (27), consistent with a crucial role for oxidants in the regulation of pulmonary vasotone.

Nox4 up-regulation during PH has been observed multiply at both the messenger RNA (mRNA) and protein levels (4, 20, 21, 33, 40), but little is known about the downstream targets of the H_2_O_2_ generated by this enzyme (21). A large body of literature describes detrimental effects of Nox4 in PH (21, 22, 41, 42). For example, inhibition of Nox4 by VCC588646, VCC202273, or GKT136901 reduced PASMC proliferation in vitro, and vascular remodeling together with RV hypertrophy in monocrotaline-treated rats (22), consistent with a causative role for Nox4 in PH. However, the specificity and isoform selectivity for many of these inhibitors have been questioned (43), and thus the protection observed may result from a widespread inhibition of superoxide-producing Nox isoforms, rather than Nox4 alone. Indeed, a recent report demonstrated that constitutive or inducible Nox4 knock-out mice develop a similar PH phenotype to WTs when subjected to chronic hypoxia for 21 d (44). Therefore, Nox4 up-regulation may serve as a protective rather than a detrimental mechanism. Nox4-mediated cysteine oxidation of K_V_1.5 channels leads to the inhibition of the channel activity, sustained depolarization, and pulmonary vasoconstriction observed during the pathogenesis of hypoxia-induced PH (21). In contrast, another group showed that K_V_1.5 oxidation induces activation of these channels, which couples to vasodilation (rather than vasoconstriction) in response to H_2_O_2_ (45). Oxidants also induce disulfide PKGIα (8, 10, 30), which significantly mediates vasodilation by H_2_O_2_, including via phosphoactivation of large-conductance potassium channels (46). It is likely that chronic hypoxia results in oxidation of multiple other proteins, but studying the “redox-dead” PKGI KI allowed a specific role for oxidation of this kinase in adaptation to PH to be defined (7, 47).

The expression of CSE enzyme was also increased during hypoxia. CSE is known for its generation of the vasorelaxant H_2_S (48), while this enzyme may also directly generate cysteine persulfides from cystine (49). The term “persulfide” is defined here as all molecular species containing more than one sulfur atom in each low-molecular-weight and protein/peptidyl thiol moiety. H_2_S can additionally be oxidized to the persulfides by the product of SOD3 and Nox4, namely H_2_O_2_ (9, 50), or directly by Cu/Zn SOD (51). These persulfide species cause vasodilation and blood pressure lowering by promoting disulfide PKGIα (9). Thus, the increased expression of SOD3, Nox4, and CSE would provide an integrated mechanism that limits dysfunction during PH by generating oxidant species that couple to vasodilation by increasing disulfide PKGIα. Such vasodilation would reduce pulmonary and RV pressure to limit the progressive adverse remodeling, consistent with the disease-limiting effects of therapies that enhance cGMP-dependent activation of PKGI (52–54). Therefore, protein oxidation, as observed in idiopathic PAH (55), may provide beneficial, adaptive mechanisms, as opposed to the solely deleterious role it has historically been associated with. It is notable that the increased disulfide PKGIα, as well as the Nox4 and CSE enzymes that likely contribute to oxidation of the kinase, are observed in samples from humans with PAH (Fig. 1*D*).

### Alteration of Reactive Oxygen and Sulfur Species Metabolome During Chronic Hypoxia.

**Fig. 2.**
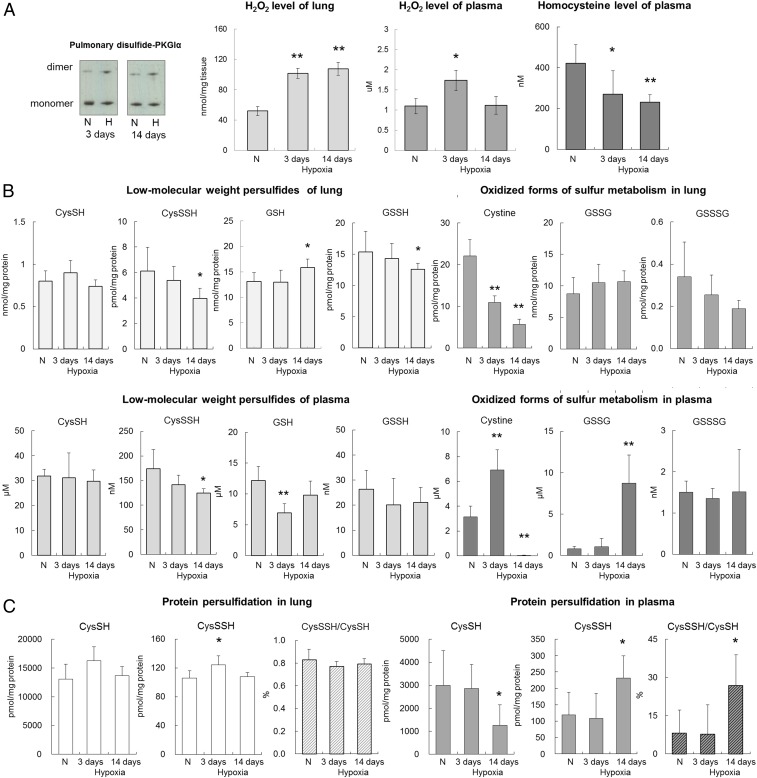
Reactive oxygen and sulfur species level in lung and plasma of mice subjected to chronic hypoxia. (*A*) Representative disulfide PKGIα increase in lungs of WT mice subjected to either normoxia or chronic hypoxia for 3 and 14 d. (*B*) Low-molecular-weight persulfides and oxidized forms of sulfur metabolism in lung and plasma of WT mice subjected to either normoxia or chronic hypoxia for 3 and 14 d. Thiol and hydropersulfide-containing compound were alkylated with tyrosine and a hydroxyphenyl-containing derivative, β-(4-hydroxyphenyl)ethyl iodoacetamide (HPE-IAM), and their HPE-IAM adducts were quantified by liquid chromatography-electrospray ionization-tandem mass spectrometry (LC-ESI-MS/MS). (*C*) Protein persulfidation in lung and plasma of WT mice subjected to either normoxia or chronic hypoxia for 3 and 14 d. Protein-bound hydropersulfides were alkylated with HPE-IAM, and pronase digest samples were quantitatively analyzed by LC-ESI-MS/MS. *P < 0.05, **P < 0.01 versus normoxia; *n* = 6 to 8 per group. In some cases, the aspect ratio of the original immunoblots was altered to enable a concise multipanel figure with a consistent presentation style; the original uncropped representative images of these immunoblots are also available in *SI Appendix*, Fig. S10.

To further explore the molecular nature of the species that mediate PKGIα oxidation, reactive oxygen and sulfur metabolites were analyzed in lungs or plasma of mice subjected to chronic hypoxia for 3 and 14 d. Hypoxia increased pulmonary disulfide PKGIα levels (Fig. 2*A*), substantiating the observations shown in Fig. 1. A one-step fluorescence-based Amplex Red assay was employed to assess H_2_O_2_ abundance in these samples, as it is a product of both Nox4 and SOD3. We observed a time-dependent increase of H_2_O_2_ in lungs of mice subjected to hypoxia, whereas plasma H_2_O_2_ was only elevated after 3 d (Fig. 2*A*). This was anticipated and rationalized by the concomitantly increased Nox4 and SOD3 expression in pulmonary arteries after 3 d or 14 d of hypoxia (Fig. 1 *A* and *B*). The ROS-generating activity of Nox4 is regulated primarily by its expression (56–58); in addition, both mRNA and protein levels of Nox4 can be up-regulated via hypoxia-inducible factor 1α (33). Therefore, it is likely that H_2_O_2_ elevation in hypoxic lungs is, at least partially, a result of increased protein abundance and activity of this oxidase. Overall, the elevated amounts of H_2_O_2_ observed in hypoxic tissues are consistent with increased expression and activity of both Nox4 and SOD3, but other ROS-producing enzymes may also contribute.

Next, the sulfur metabolites were analyzed by mass spectrometry, as before (49, 59). Hypoxia induced a time-dependent reduction in plasma homocysteine (Fig. 2*A*), which may reflect the increased expression and activity of pulmonary CSE, consuming this as a substrate. Another H_2_S producing enzyme, namely cystathionine β-synthase, can also metabolize homocysteine (60); however, we could not detect this protein in the lung. Reactive sulfur intermediates, such as low-molecular-weight persulfides (RS_n_H and RSS_n_R, *n* > 1), as well as total protein persulfidation were assessed (Fig. 2 *B* and *C* and *SI Appendix*, Fig. S3). These sulfur intermediates have inherent chemical properties that confer reactivity to different biological targets (49, 59), and are anticipated to alter the thiol redox state of proteins, including PKGIα. Cysteine (CysSSH) and glutathione persulfides (GSSH) were time-dependently decreased in both lungs and plasma, although the change in GSSH levels failed to reach statistical significance in plasma (Fig. 2*B*). This is notable, as these hydropersulfide species are superior reductants that are capable of rapid neutralizing reactions with ROS, as well as the reduction of disulfide-containing molecules (49, 61), including those on PKGI. Other sulfur derivatives such as HS^−^, HSS^−^, and thiosulfates (HS_2_O_3_^−^) were also decreased in the plasma and lung in hypoxia (*SI Appendix*, Fig. S3). Reduction of such reducing equivalents will favor accumulation of oxidized products, and is consistent with the increased disulfide PKGIα present in the pulmonary system during hypoxia. It was notable that lung cystine was decreased in a time-dependent manner during hypoxia, while its plasma level was hardly detectable at 14 d of hypoxia, after a transient increase at 3 d (Fig. 2*B*). Cystine is an alternative substrate for CSE that can be utilized by this enzyme specifically during oxidative stress (49). Therefore, these data further corroborate an increase in systemic CSE activity, and are consistent with increased oxidative stress and higher amounts of disulfide PKGIα during hypoxia.

Thus, the abundance of some reactive sulfur species is attenuated in hypoxia-induced PH. Interestingly, a decrease in reactive persulfide species accompanied by increased CSE expression was recently reported in the lungs of patients with chronic obstructive pulmonary disease (62). Such lower amounts of hydropersulfides may appear counterintuitive to increased CSE activity (61). A likely explanation of this is that the sulfur species produced by CSE are eliminated by H_2_O_2_ or other endogenous electrophiles accumulating in the hypoxic tissue. Indeed, oxidized glutathione was increased in plasma (Fig. 2*B*), while no increase in plasma H_2_O_2_ was observed after 14 d of hypoxic exposure (Fig. 2*A*), further supporting this suggestion. Furthermore, protein-bound persulfides (CysSSH/CysSH) were increased in plasma after 14 d of hypoxia (Fig. 2*C*). Enhanced protein persulfidation in plasma may represent a compensatory induction of persulfide biosynthesis in response to the sulfide consumption during hypoxia. Although the exact underlying mechanisms still remain unclear, these observations are consistent with systemic oxidative stress, including within the pulmonary system.

### Effect of CSE Inhibition and Polysulfide Donors on Hypoxia-Induced PH.

**Fig. 3.**
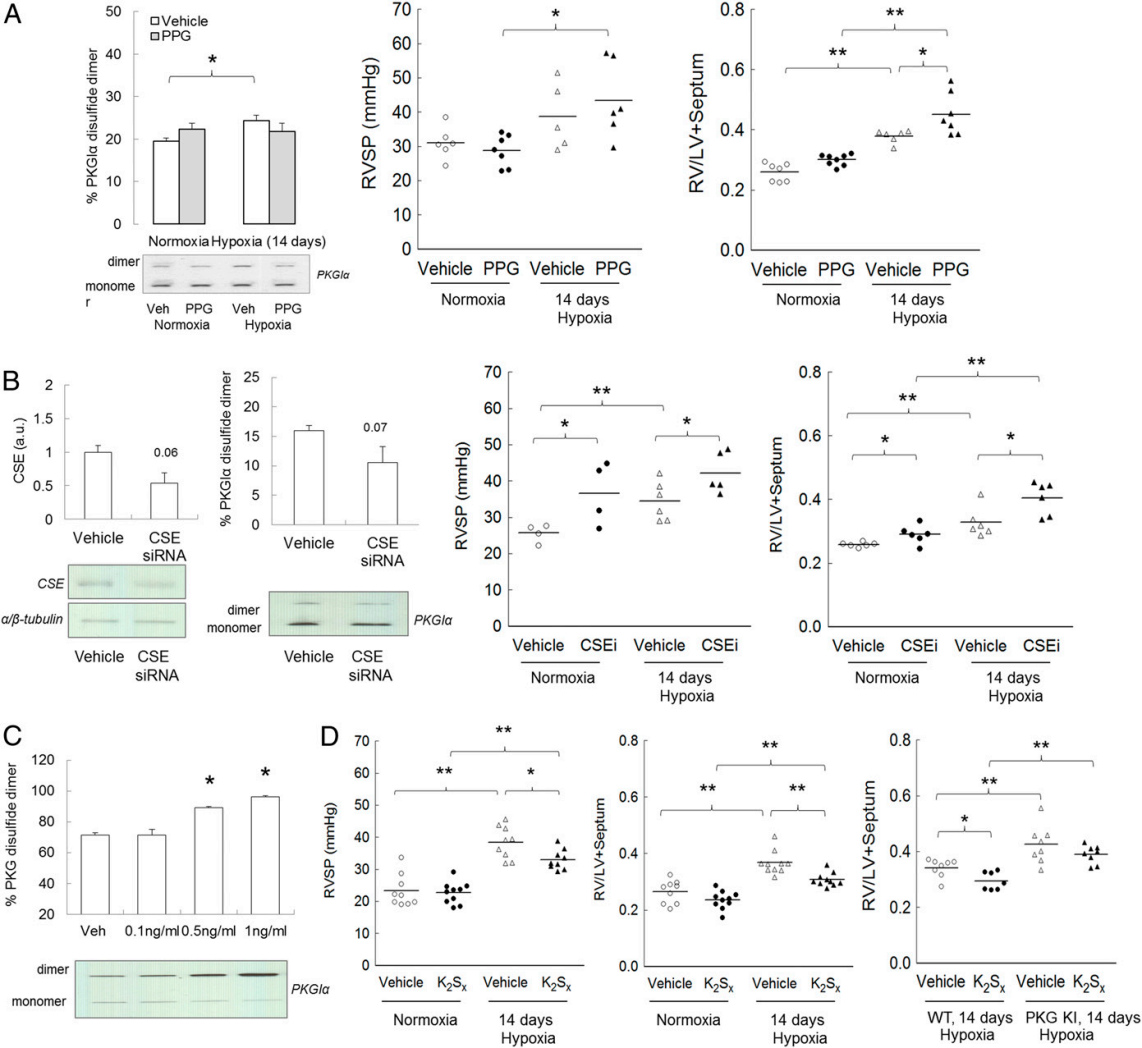
Effect of CSE inhibition and polysulfides in hypoxia-induced PH. (*A*) Pulmonary disulfide PKGIα expression, RV pressure, and RV to left ventricle + septum (LV+S) ratio in C57BL/6 mice subjected to either normoxia or chronic hypoxia for 14 d with or without CSE inhibitor l-PPG (50 mg/kg/d). (*B*) CSE protein expression and disulfide PKGIα level in lungs of mice treated with CSE siRNA (1.3 mg⋅kg^−1^⋅d^−1^); RV pressure and RV to LV+S ratio in WT mice subjected to either normoxia or chronic hypoxia for 14 d with or without CSE siRNA (1.3 mg⋅kg^−1^⋅d^−1^). (*C*) Disulfide PKGIα formation in response to persulfides donor K_2_Sx treatment in human pulmonary artery smooth muscle cells. (*D*) RV pressure and RV to LV+septum ratio in C57BL/6 mice subjected to either normoxia or chronic hypoxia for 14 d with or without persulfides donor K_2_Sx (2 mg⋅kg^−1^⋅d^−1^); RV to LV+S ratio in WT or KI mice subjected to chronic hypoxia for 14 d with or without K_2_Sx (2 mg⋅kg^−1^⋅d^−1^). *P < 0.05, **P < 0.01 versus control; *n* = 5 to 7 per group. In some cases, the aspect ratio of the original immunoblots was altered to enable a concise multipanel figure with a consistent presentation style; the original uncropped representative images of these immunoblots are also available in *SI Appendix*, Fig. S10.

To experimentally test the importance of CSE in limiting PH during chronic hypoxia, C57BL/6 mice were subjected to chronic hypoxia for 14 d with or without l-propargylglycine (l-PPG)—a pharmacological inhibitor of this enzyme; 14 d of hypoxia, albeit not when l-PPG was also present, increased pulmonary disulfide PKGIα levels compared with normoxia (Fig. 3*A*). Moreover, l-PPG treatment of mice subjected to hypoxia increased RV pressure and hypertrophy compared with vehicle-treated hypoxic mice (Fig. 3*A*). CSE expression was decreased in WT or KI mice by administering small interfering RNA (siRNA) to them in vivo. This silencing approach, which was initially validated in mouse PASMCs (*SI Appendix*, Fig. S4*A*), decreased lung CSE protein by nearly 50%, with a trend toward decreased disulfide PKGIα in the same samples (Fig. 3*B*). As with inhibition of CSE by l-PPG, siRNA-induced knockdown of this enzyme increased RV pressure and hypertrophy in WT mice compared with vehicle-treated controls subjected to normoxia or hypoxia (Fig. 3*B*). There was no effect of CSE siRNA on RV systolic pressure (RVSP) or RV hypertrophic remodeling in the KI mice (*SI Appendix*, Fig. S4*B*). These observations are consistent with the causal role of CSE-derived persulfide species that facilitate disulfide PKGIα and limit PH and the consequent dysfunction.

To test this concept further, mice were exposed to potassium polysulfides (persulfides) or sodium hydrosulfide, interventions that previously induced disulfide PKGIα (9). Once again, potassium polysulfide induced PKGIα oxidation, this time in human PASMCs (Fig. 3*C*), and either compound limited RV pressure increases and hypertrophy in C57BL/6 mice subjected to hypoxia (Fig. 3*D* and *SI Appendix*, Fig. S4*C*), consistent with its vasodilatory role. Notably, the protection provided to WT mice by polysulfides was less evident in the KI mice (Fig. 3*D*), further strengthening the rationale that disulfide PKGIα formation in the lung during hypoxia serves as a protective mechanism. These observations strike a chord with the inverse correlation between plasma H_2_S level and pulmonary arterial pressure observed in humans with PAH (63).

### “Redox-Dead” Cys42Ser PKGIα KI Mice Develop More Severe Hypoxia-Induced PH Phenotype.

**Fig. 4.**
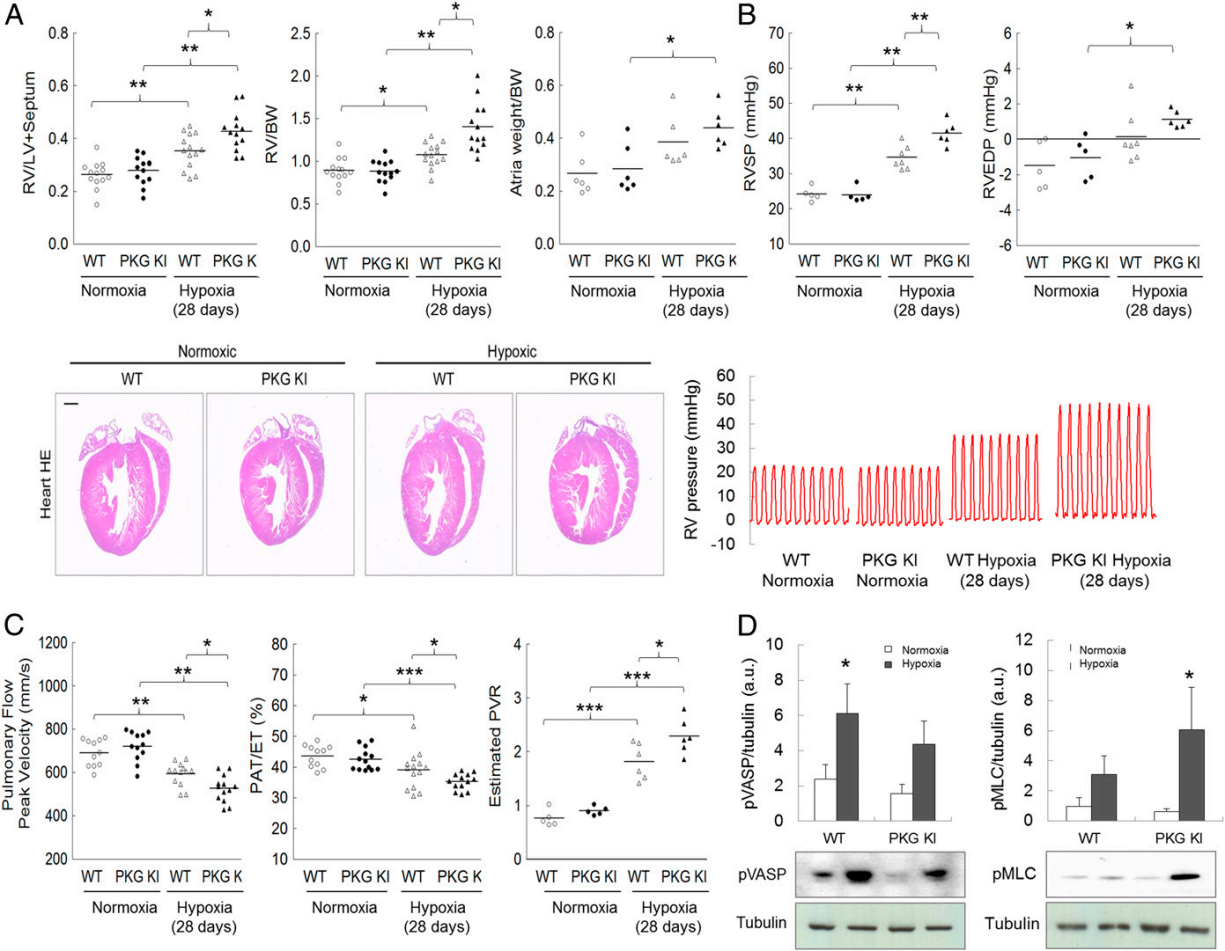
“Redox-dead” Cys42Ser PKGIα KI mice subjected to chronic hypoxia develop potentiated PH. (*A*) RV to LV+S ratio, RV to BW ratio, atria weight to BW ratio, and representative four-chamber view micrographs from hematoxylin eosin staining (H&E) stained heart sections, and (*B*) RVSP, RV end diastolic pressure (RVEDP), and representative RV pressure tracings from WT or PKG KI mice subjected to either normoxia or chronic hypoxia for 28 d. (*C*) Changes in pulmonary flow peak velocity, pulmonary acceleration time/pulmonary ejection time (PAT/ET), and estimated PVR in WT or KI mice subjected to either normoxia or chronic hypoxia for 28 d. (*D*) Phosphorylated MLC (pMLC) and phosphorylated VASP (pVASP) protein expression in lungs from WT or KI mice subjected to either normoxia or chronic hypoxia for 28 d. *P < 0.05, **P < 0.01, ***P < 0.001 versus control or respective WT; *n* = 12 to 14 per group, or, in some experiments, *n* = 5 to 7 per group. In some cases, the aspect ratio of the original immunoblots was altered to enable a concise multipanel figure with a consistent presentation style; the original uncropped representative images of these immunoblots are also available in *SI Appendix*, Fig. S10.

The role of disulfide PKGIα as an adaptive mechanism during hypoxic PH was examined in more depth by comparing disease progression in WT versus KI mice. KI mice showed potentiated increases in RV hypertrophy and pressure (Fig. 4 *A* and *B*) compared with WT during hypoxia. This potentiated dysfunction in the KIs that cannot form the targeting and activating disulfide PKGIα during hypoxia was further evidenced by an exacerbated decline in pulmonary vascular blood flow indexes, together with a higher pulmonary vascular resistance (PVR) (Fig. 4*C*). Cardiac function decline was moderate in both genotypes (*SI Appendix*, Fig. S5).

Phosphorylation of vasodilator-stimulated phosphoprotein (VASP) was decreased, while myosin light chain (MLC) phosphorylation was increased in lungs (Fig. 4*D*) and pulmonary arteries (*SI Appendix*, Fig. S6*A*) of the KI compared with WT during hypoxia. Lack of VASP phosphorylation in the KIs is consistent with deficient disulfide PKGIα targeting, and was observed previously (10). Phosphorylated MLC status is modulated by MLC kinase and by MLC phosphatase, the activity of which is phosphoregulated by PKGIα (64, 65). Disulfide PKGIα is anticipated to activate MLC phosphatase which, in turn, dephosphorylates MLC and enables smooth muscle relaxation (64). The Cys42Ser PKGIα KIs are likely deficient in this activity, resulting in higher MLC phosphorylation. This may be consistent with Cys42 oxidation targeting PKGIα, which is plausible given the disulfide occurs in the middle of the N-terminal leucine zipper of the kinase that binds a similar zipper in MLC phosphatase (47, 64). The enhanced phosphorylation of MLC in the KI during hypoxia is in agreement with enhanced vasoconstrictory kinase activation (66, 67), with an inability to suitably activate the disulfide PKGIα-dependent activation of MLC phosphatase that enables compensatory vasodilation in WT.

### H_2_O_2_-dependent Vasodilation Is Impaired in “Redox-Dead” Cys42Ser PKGIα KI Pulmonary Arteries.

Disulfide PKGIα contributes to blood pressure homeostasis, being a component of endothelium-derived hyperpolarizing factor-dependent vasodilation (8, 47). It decreases calcium concentration in vascular smooth muscle cell, and mediates oxidant-induced vasodilation (68). Since PASMCs are abundant in PKGIα and its disulfide-dimerized form, it was rational to test whether this mechanism is preserved in pulmonary vessels. First- or second-order pulmonary arteries from the KIs demonstrated impaired vasodilatory responses to H_2_O_2_ compared with the WT, despite equal constriction to the pressor agonist U-46619 in each genotype (*SI Appendix*, Fig. S6*B*). Isolated perfused mouse lung was next employed to test vascular responses in pulmonary resistance vessels. Potentiated pressor agonist-induced constriction and deficient pulmonary vasodilatory responses to H_2_O_2_ were observed in the perfused lungs from the KI mice, compared with the WT (*SI Appendix*, Fig. S6*C*), further supporting the crucial vasodilatory role of disulfide PKGIα in pulmonary circulation. This is consistent with a global role for PKGIα oxidation in vasodilation (8, 11).

Disulfide PKGIα formation transduces increased abundance of oxidants to vasodilation in the systemic circulation (8, 10), with the same basic events clearly in operation in the pulmonary system. This oxidative activation of PKGIα occurs in the pulmonary tissues of WT mice during chronic hypoxia for 28 d, and the evidence presented thus far indicates this offsets the hypertension in airway blood vessels that occurs at this time. This adaptive mechanism that enhances PKGIα activity to trigger vasodilation lowers RV pressure, thus reducing the associated RV hypertrophic remodeling. It is plausible that interventions that increase disulfide PKGIα may be therapeutic, limiting progression to heart failure in PAH patients.

Sustained pulmonary vasoconstriction is influenced by endothelial release of vasoactive mediators and calcium, as well as by changes in MLC phosphorylation (3). Protein kinase A (PKA) is another cyclic nucleotide-dependent kinase that, like PKGIα, is also redox-regulated by interprotein disulfide bond formation (69). PKA plays a role in opposing vasoconstriction by phosphorylating targets that reduce intracellular calcium concentration and promote relaxation (1, 64). Redox-dead Cys17Ser PKARIα KI mice which cannot be disulfide-activated (70) have increased aortic vascular reactivity to vasopressor phenylephrine and deficient H_2_O_2_-dependent relaxation (71). We subjected Cys17Ser PKARIα KI mice to chronic hypoxia, but, in contrast to the Cys42Ser PKGIα KI, found no differences in RV hypertrophy between the genotypes (*SI Appendix*, Fig. S7). These observations are consistent with a specific adaptive role for disulfide PKGIα in the lung in hypoxic PH scenario.

### “Redox-Dead” Cys42Ser PKGIα KI Mice Develop Potentiated Pulmonary EndoMT During Hypoxia-Induced PH.

**Fig. 5.**
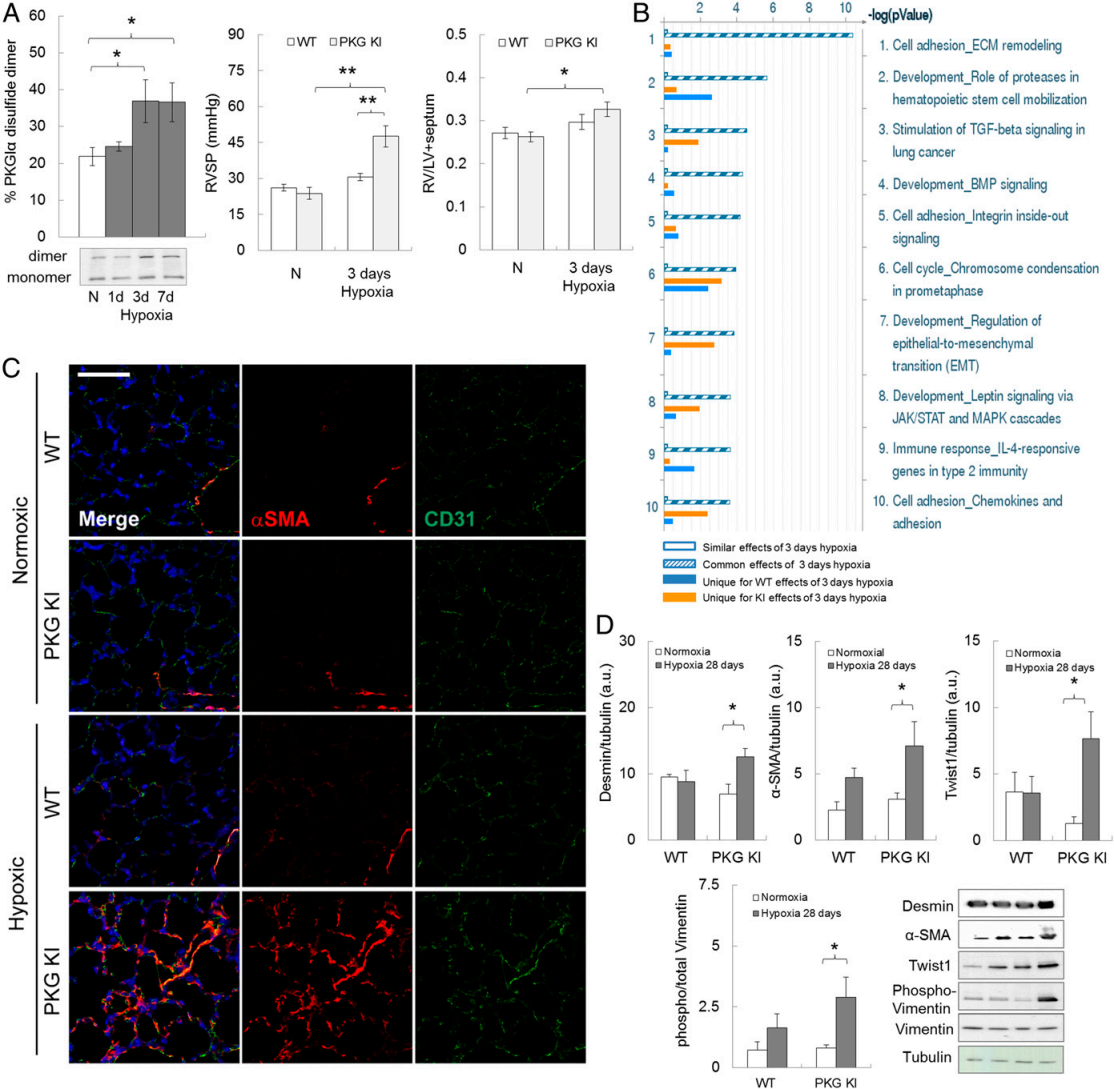
Enhanced pulmonary vascular growth signaling and EndoMT in redox-dead Cys42Ser PKGIα KI mice subjected to chronic hypoxia. (*A*) Pulmonary disulfide PKGIα level in C57BL/6 mice subjected to hypoxia for 1, 3, and 7 d; RVSP and RV to LV+S ratio from WT or PKG KI mice subjected to short-time chronic hypoxia for 3 d. *P < 0.05, **P < 0.001 versus control or WT; *n* = 6 to 8 per group. (*B*) Unbiased pathway analysis of processes with the largest number of alterations in gene expression in lungs of WT or KI mice subjected to 3 d of hypoxia compared with normoxic animals, listed in descending order. (*C*) Representative confocal images in lung sections from WT or KI mice subjected to chronic hypoxia for 28 d, stained simultaneously with nuclear (DAPI, blue), smooth muscle (α-SMA, red), and endothelial (CD31, green) markers. (Scale bar, 50 μm.) (*D*) Desmin, α-SMA, Twist-1, and phospho-vimentin protein expression in lungs from WT or KI mice subjected to chronic hypoxia for 28 d. *P < 0.05 versus control; *n* = 6 to 8 per group. ECM, extracellular matrix; TGF, transforming growth factor; BMP, bone morphogenetic protein; JAK/STAT; janus kinase/signal transducer and activator of transcription proteins; MAPK, mitogen-activated protein kinase; α-SMA, α-smooth muscle actin; CD31, cluster of differentiation 31; WT, wild type mice; PKG KI, “redox-dead” Cys42Ser PKGIα KI mice. Unique effect is gene changes which are unique to either the WT or KI group. Common effects are gene changes that are common to both the WT as well as the KI group. Similar effects are gene changes which are neither common nor unique to WT or KI groups. This terminology reflects the definitions in the Metalcore Training Manual (Version 5.0). In some cases, the aspect ratio of the original immunoblots was altered to enable a concise multipanel figure with a consistent presentation style; the original uncropped representative images of these immunoblots are also available in *SI Appendix*, Fig. S11.

Smooth muscle cell proliferation contributes to excessive muscularization of pulmonary vessels during PH (2, 3). PKGI not only modulates vasotone and the pressure within arteries but also continues to emerge as an important player in cell differentiation, growth, proliferation, and cancer progression (72–74). RV hypertrophic remodeling and pressure were assessed in WT and KI mice subjected to hypoxia for 3 d, a time when pulmonary disulfide PKGIα was elevated (Figs. 1*A* and 5*A*). WT mice developed no RV hypertrophic remodeling at this time point, and the RV pressure was similar to that in normoxic mice, meaning that PH was reversible in the WT upon return to normoxia—as occurs, for practical reasons, when RV pressure is measured (Fig. 5*A*). Interestingly, the PKGI KI mice developed a larger increase in RV pressure and hypertrophy, compared with the WTs, consistent with a more pronounced and sustained pulmonary vasoconstriction—perhaps marking the start of remodeling processes in the KI due to hypoxia. It is likely that the disulfide PKGIα is crucial in the pulmonary circulation even after 3 d of hypoxic exposure, by limiting increases in PAP and RVSP, and this is likely why the KI mice that cannot form the disulfide in PKGI develop a larger or more sustained pressure increase.

Three days of hypoxia was therefore considered a logical time point to monitor changes in gene expression, as this is before structural remodeling in the WT had occurred, in an attempt to define additional events that are important in the pathogenesis of hypoxic pulmonary disease. Thus, a transcriptomic screen using an Affymetrix microarray was performed on lungs from WT or KI mice subjected to normoxia or hypoxia for 3 d. Pathway analysis of these mRNA expression abundance data revealed an up-regulation of progrowth, extracellular matrix remodeling and endothelial-to-mesenchymal transition (EndoMT) cellular signaling pathways in the KI compared with the WT after 3 d of hypoxia (Fig. 5*B*). This was notable, as EndoMT recently emerged as an important regulator of pulmonary vascular remodeling in rodent models of PH and human disease (75).

Affymetrix microarray mRNA analysis was performed on the whole lung; therefore, it was necessary to establish whether the increased growth and EndoMT were evident in pulmonary blood vessels. Increased coexpression of α-smooth muscle actin (α-SMA) and cluster of differentiation 31 (CD31) in lung endothelial cells of the KI mice subjected to hypoxia was prominent compared with that measured in WT (Fig. 5*C*). Protein expressions of α-SMA and desmin, as well as the EndoMT transcriptional regulator Twist-1 and phosphorylated Vimentin (75) (Fig. 5*D*), were increased in the lungs of the KI to a greater extent than those of WT following hypoxia. The KI mice subjected to 28 d of hypoxia demonstrated significantly exacerbated pulmonary vascular muscularization compared with WT exposed to the same intervention, as evidenced by a greater accumulation of α-SMA expressing cells in pulmonary vessels (*SI Appendix*, Fig. S6*D*). It is plausible that increased disulfide PKGIα during hypoxia may prevent pulmonary vascular muscularization, possibly by impairing EndoMT. Whether this mechanism serves to alleviate pressure and PVR, perhaps independently of the disulfide PKGIα pressure-lowering role, remains to be definitively elucidated.

In summary, disulfide PKGIα accumulates during chronic hypoxia in mouse and man, likely due to the accumulation of H_2_O_2_, glutathione disulfide, and protein-bound persulfides under these conditions. Depletion of superreducing persulfide species in mouse hypoxic tissues may also contribute to disulfide PKGIα abundance, which serves as an endogenous, adaptive redox signaling mechanism that limits PH to attenuate RV hypertrophy and disease progression. Disulfide PKGIα may also prevent the progression of EndoMT and so limit adverse pulmonary vascular remodeling. Pharmacological interventions that enhance disulfide PKGIα levels, such as polysulfides as demonstrated herein, may provide a novel therapeutic strategy to combat disease resulting from PH.

## Materials and Methods

### Animals, Induction of Hypoxic Pulmonary Hypertension, and Treatment.

All animal procedures were performed in accordance with the Home Office Guidance on the Operation of the Animals (Scientific Procedures) Act 1986 in the United Kingdom and were approved by the King’s College Animal Welfare and Ethical Review Body. Mice constitutively expressing PKGIα Cys42Ser were produced on a pure C57BL/6 background by Taconic Artemis as described (8, 76) and bred on-site. Age- and body weight-matched WT or PKGIα Cys42Ser KI male offspring were used in most of the studies. In some experiments, age- and body weight-matched adult C57BL/6 male mice were purchased from Charles River, as highlighted in more detail in *Results and Discussion*. Animals had ad libitum access to standard chow and water and were kept in specific pathogen-free conditions under a 12-h day/night cycle at 20 °C and 60% humidity before hypoxic exposure. Hypoxic PH was induced by exposing mice to normobaric hypoxia (10% of inspired O_2_) in a large ventilated chamber (Biospherix, Ltd) (*SI Appendix*, Fig. S1). The CO_2_ level was monitored continuously with CO_2_ meter and soda lime. Fresh cage, water, and food changes were performed once every 7 d to 10 d for all of the animals. Additional materials and procedures can be found in *SI Appendix*, *SI Materials and Methods*.

### Study Approval.

All animal procedures were performed in accordance with the Home Office Guidance on the Operation of the Animals (Scientific Procedures) Act 1986 in the United Kingdom and were approved by the King’s College Animal Welfare and Ethical Review Body. The protocol of the study using human samples was approved by the Ethics Committee of the Justus-Liebig-University School of Medicine (No. 111/08 and 58/15). Informed consent was obtained in written form from each subject.

## Supplementary Material

Supplementary File
