# Supplementary material for: Oxidation of PKGIα mediates an endogenous adaptation to pulmonary hypertension
Source: Proc Natl Acad Sci U S A. 2019 Jun 11;116(26):13016–25. doi: 10.1073/pnas.1904064116 (PMC6601250; doi:10.1073/pnas.1904064116)
Supplement: Supplementary File [file pnas.1904064116.sapp.pdf]

PNAS
www.pnas.org

Supplementary Information for

Oxidation of PKGIα mediates an endogenous adaptation to pulmonary hypertension

Olena Rudyk[a,1], Alice Rowan[a], Oleksandra Prysyazhna[a], Susanne Krasemann[b], Kristin Hartmann[b], Min Zhang[a], Ajay M. Shah[a], Clemens Ruppert[c], Astrid Weiss[d], Ralph T. Schermuly[d], Tomoaki Ida[e], Takaaki Akaike[e], Lan Zhao[f], and Philip Eaton[a,1]

[a]School of Cardiovascular Medicine & Sciences, British Heart Foundation Centre of Excellence, King's College London, London, United Kingdom; [b]Institute of Neuropathology, University Medical Centre Hamburg-Eppendorf, Hamburg, Germany; [c]Universities of Giessen & Marburg Lung Center Giessen Biobank, Justus-Liebig-University Giessen, Giessen, Germany; [d]Excellence Cluster Cardio-Pulmonary System, Justus-Liebig-University Giessen, Giessen, Germany; [e]Department of Environmental Medicine and Molecular Toxicology, Tohoku University Graduate School of Medicine, Sendai, Japan; and [f]Faculty of Medicine, Department of Medicine, Imperial College London, London, United Kingdom

[1]Correspondence should be addressed to:

Olena Rudyk
School of Cardiovascular Medicine & Sciences,
BHF Centre of Research Excellence, King's College London
The Rayne Institute, St. Thomas' Hospital, London, UK
Westminster Bridge Road, SE1 7EH
Telephone: 442071888351
Fax: 442071880970
Email: olena.rudyk@kcl.ac.uk

Philip Eaton
School of Cardiovascular Medicine & Sciences,
BHF Centre of Research Excellence, King's College London
The Rayne Institute, St. Thomas' Hospital, London, UK
Westminster Bridge Road, SE1 7EH
Telephone: 442071880969
Fax: 442071880970
Email: philip.eaton@kcl.ac.uk

This PDF file includes:
Figs. S1 to S7
Supplementary Materials and Method

Fig. S1 Validation of hypoxia-induced pulmonary hypertension model in mice. (a) RV/LV+septum and RV/BW, **(b)** hematocrit and haemoglobin levels, and **(c)** pulmonary vascular flow parameters in C57BL/6 mice subjected either to normoxia or chronic hypoxia for 28 days. n=5-6/group, *P< 0.05, **P< 0.01 versus normoxia. **(d)** Representation of a Doppler mode recording of pulmonary arterial flow by echocardiography in C57BL/6 mice subjected either to normoxia or chronic hypoxia for 28 days. RV, right ventricle; LV, left ventricle; BW, body weight; Hct, haematocrit; Hb, haemoglobin; PV, pulmonary vascular; ET, pulmonary ejection time; PAT, pulmonary acceleration time; SOD, extracellular superoxide dismutase; WT, wild type mice; PKG KI, 'redox-dead' Cys42Ser PKGIα KI mice.

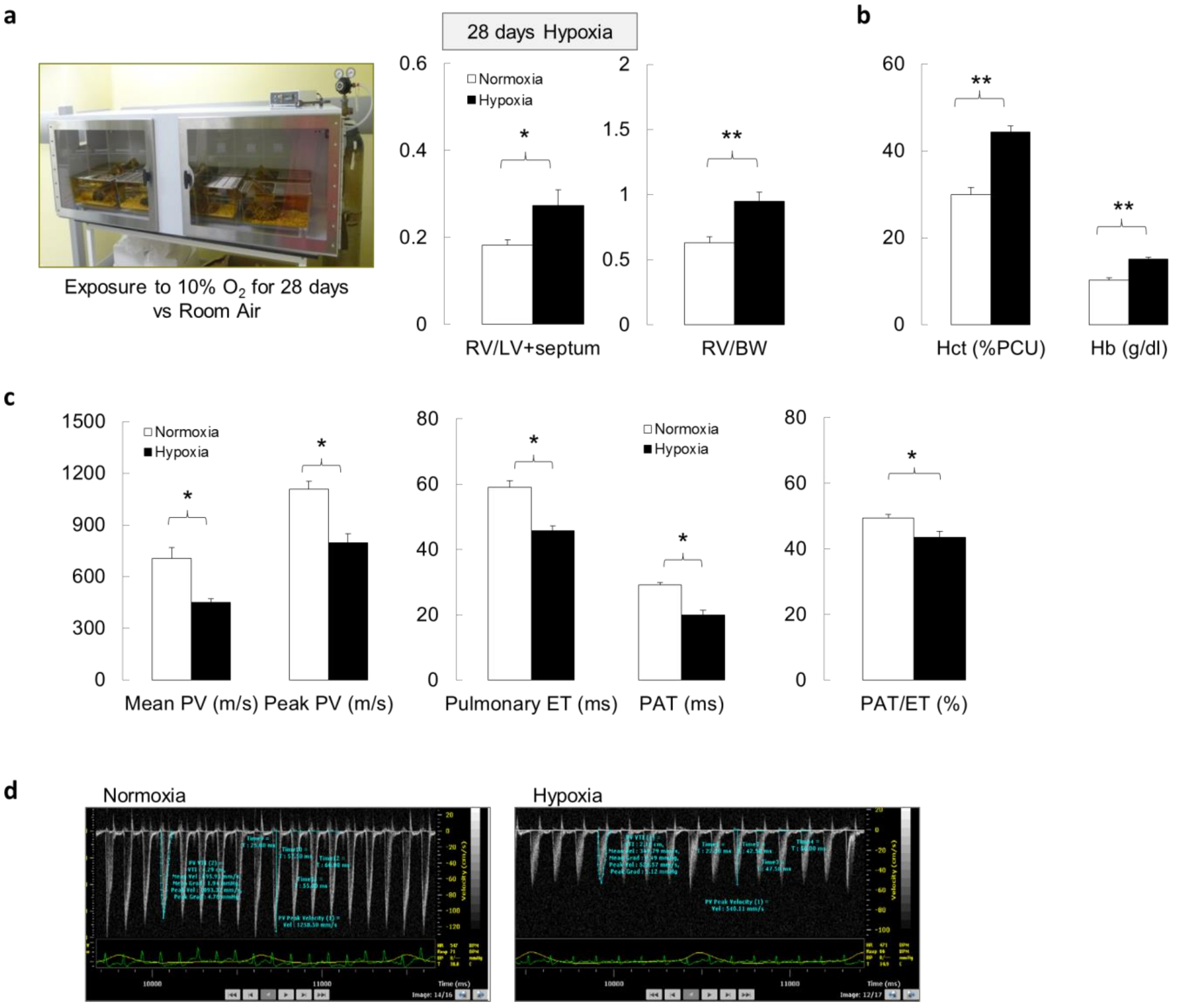

Fig. S2 Disulfide-PKGIα in the heart and SOD expression in lungs of mice subjected to chronic hypoxia. (a) Disulfide-PKGIα in RV and LV+septum of WT or Cys42Ser PKGIα knock-in (KI) mice subjected either to normoxia or chronic hypoxia for 28 days. **(b)** SOD1 and SOD2 protein expression in lungs of WT or Cys42Ser PKGIα knock-in (KI) mice subjected either to normoxia or chronic hypoxia for 28 days. **(c)** Validation of Nox4 antibodies using Nox4 knock-out lung tissues. *P< 0.05 versus Control.

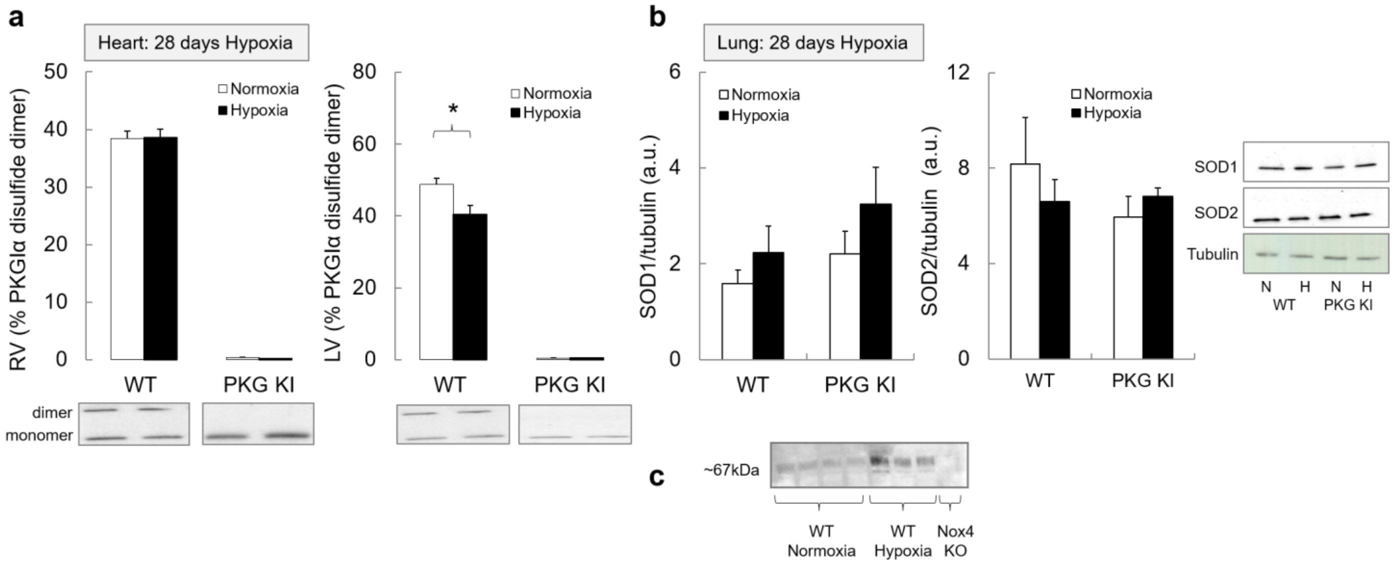

Fig. S3 Various sulfur species identified in mouse lung and plasma. Low-molecular-weight persulfides in lung **(a)** and plasma **(b)** of WT mice subjected either to normoxia or chronic hypoxia for 3 and 14 days. Hydrogen sulfide, hydrogen disulfide and thiosulfate were detected as HPE-IAM adducts (Bis-S-HPE-AM, Bis-SS-HPE-AM, HS_2O_3-HPE-AM, respectively) by LC-ESI-MS/MS. *P< 0.05, **P< 0.01 versus Normoxia.

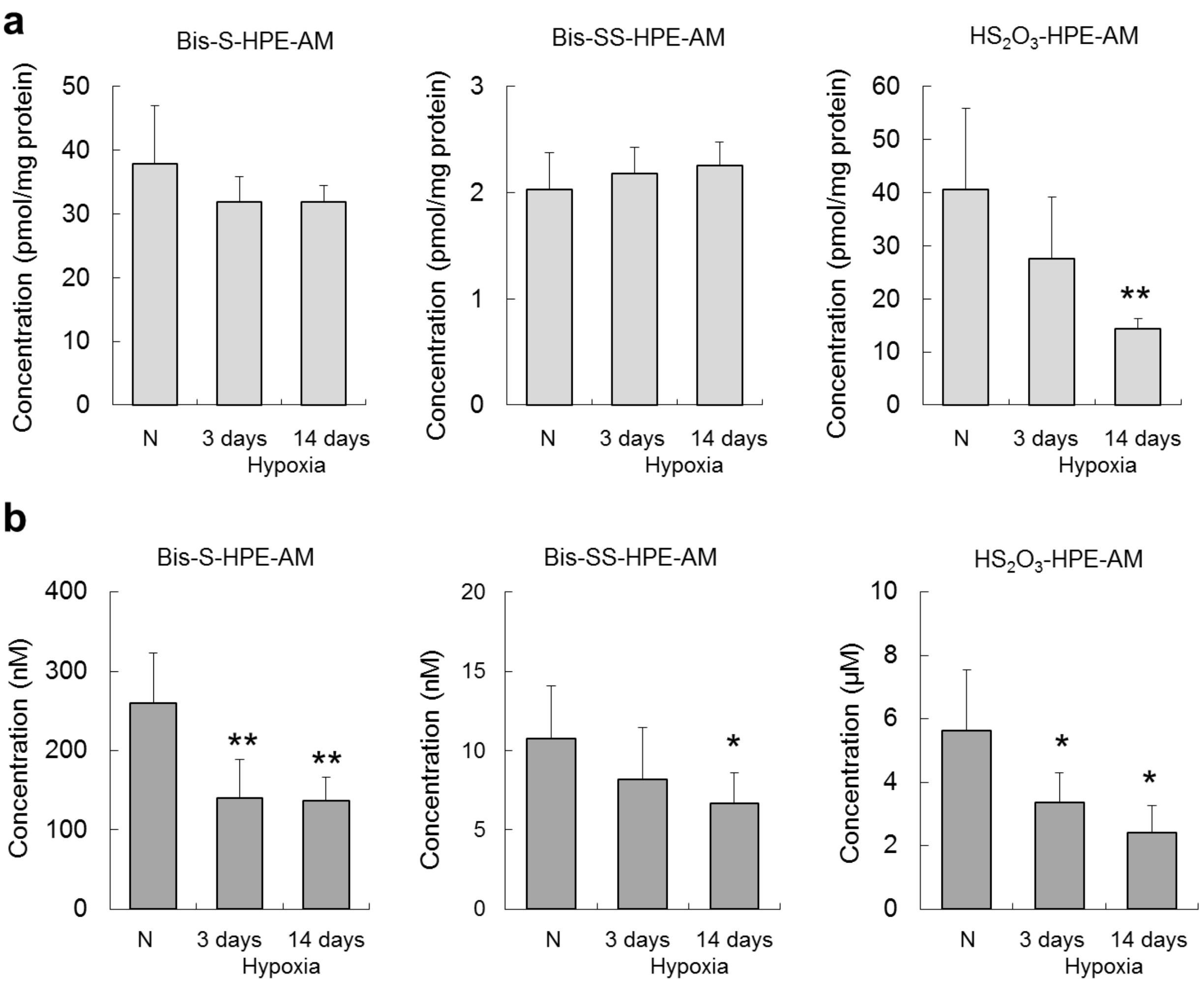

Fig. S4 Validation of siRNA CSE in PASMCs and NaHS treatment in hypoxia-induced PH. (a) CSE protein expression in PASMCs treated with various concnetrations of CSE siRNA; **(b)** RV pressure and RV to LV+septum ratio in Cys42Ser PKGIα knock-in (KI) mice subjected either to normoxia or chronic hypoxia for 14 days with or without CSE siRNA (1.3 mg/kg/day). **(c)** RV pressure and RV to LV+septum ratio in C57BL/6 mice subjected either to normoxia or chronic hypoxia for 14 days with or without H_2S donor NaHS (23 mg/kg/day). *P< 0.05, **P< 0.01 versus Control.

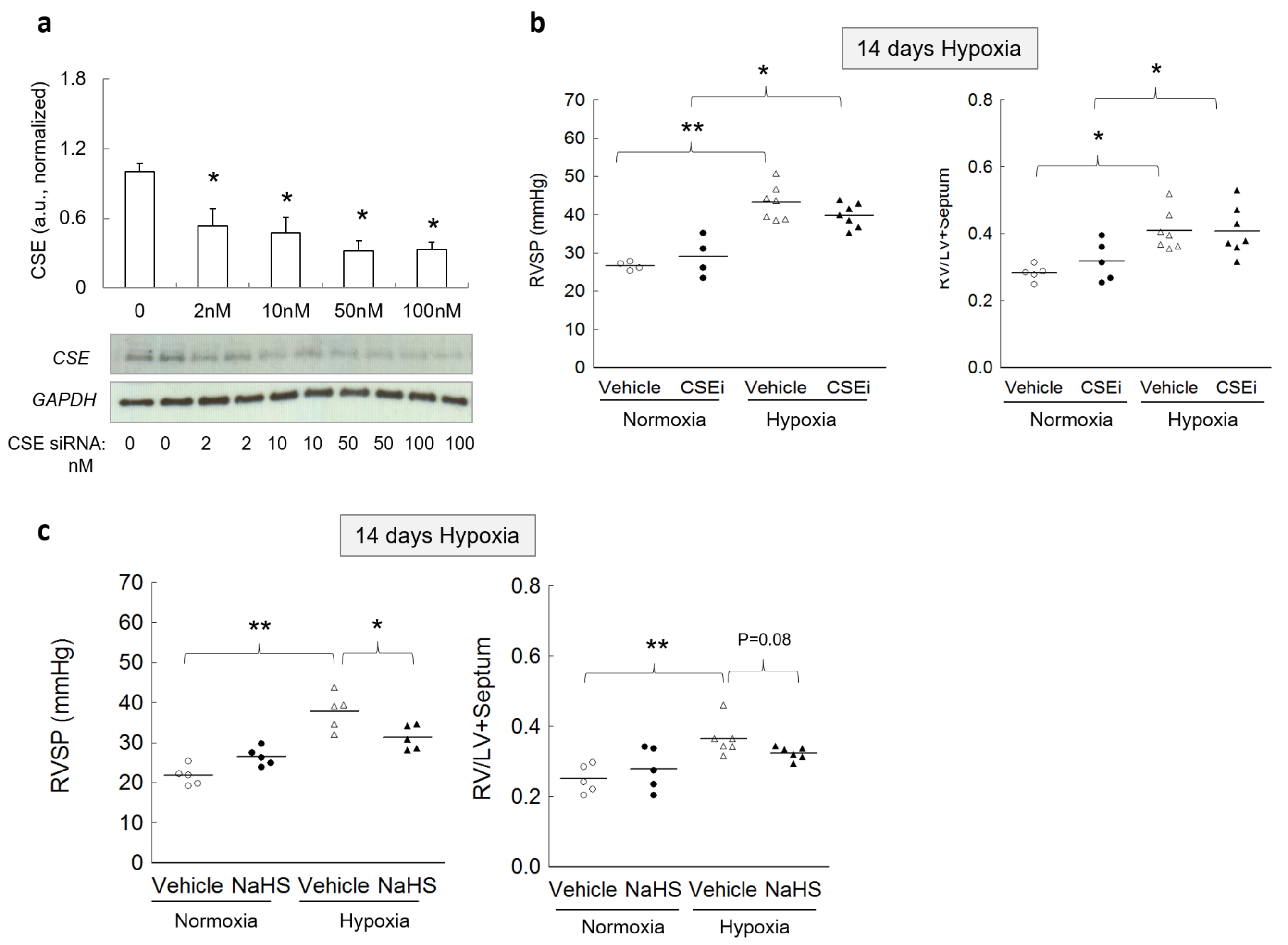

Fig. S5 Changes in left ventricular function following chronic hypoxia in 'redox-dead' Cys42Ser PKGIα KI mice. Cardiac echocardiography data from WT or KI mice subjected to chronic hypoxia for 28 days. n=12–14/group, *P< 0.05, **P< 0.01, ***P< 0.001 versus Control.

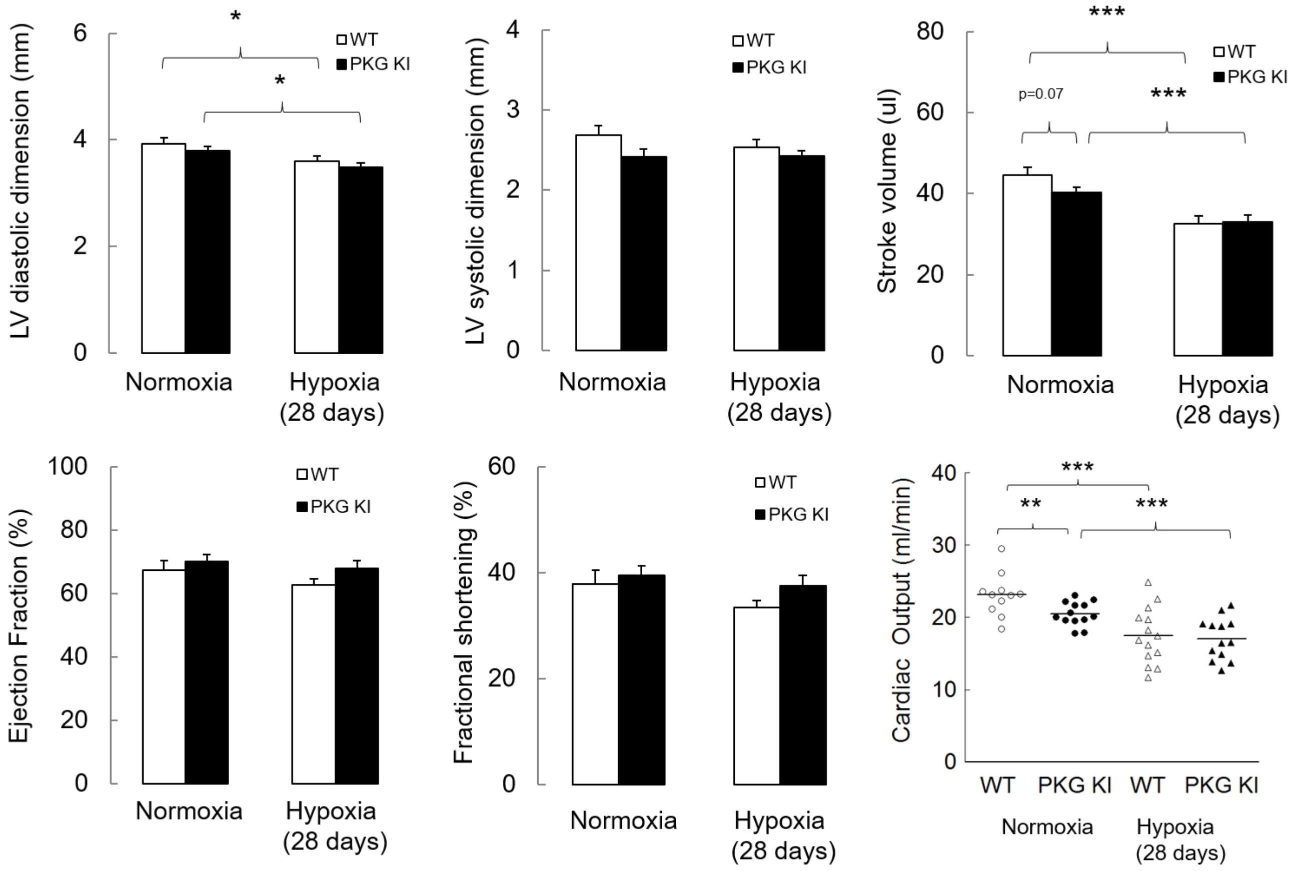

Fig. S6 Pulmonary vascular responses in 'redox-dead' Cys42Ser PKGIα KI mice. (a) pMLC and pVASP protein expression in pulmonary arteries from WT or KI mice subjected either to normoxia or chronic hypoxia for 14 days. **(b)** Vascular responses to constrictor U-46619 and vasodilator H_2O_2 in isolated pulmonary arteries and **(c)** in isolated perfused lungs from WT and 'redox-dead' Cys42Ser PKGIα KI mice under normoxia. n=6/group. **(d)** Representative micrographs from lung sections from WT or 'redox-dead' Cys42Ser PKGIα KI mice subjected to chronic hypoxia for 28 days, stained with α-SMA. Scale bar 100μM. *P< 0.05, **P< 0.01, ***P< 0.001 versus WT. WT, wild type mice; PKG KI, 'redox-dead' Cys42Ser PKGIα KI mice, α-SMA, α-smooth muscle actin.

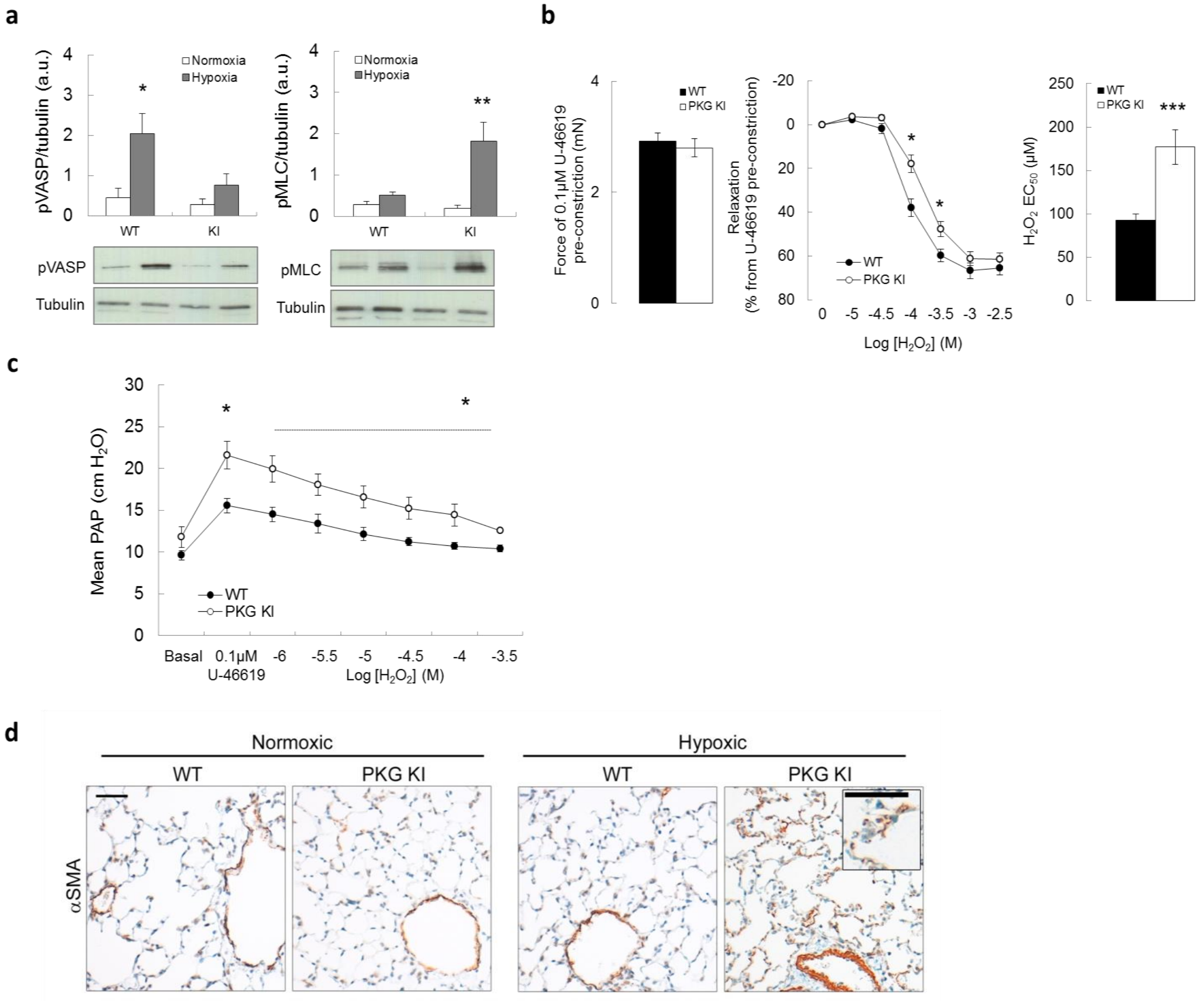

Fig. S7 RV hypertrophy in 'redox-dead Cys17Ser PKARIα KI mice subjected to chronic hypoxia. **(a)** RV to LV+S ratio, **(b)** RV to BW ratio and **(c)** LV+septum/BW ratio from WT or redox-dead PKA KI mice subjected to chronic hypoxia for 28 days. n=5–6/group, **P< 0.01 versus Control. RV, right ventricle, LV, left ventricle, BW, body weight; WT, wild type mice; PKA KI, 'redox-dead' Cys17Ser PKARIα KI mice.

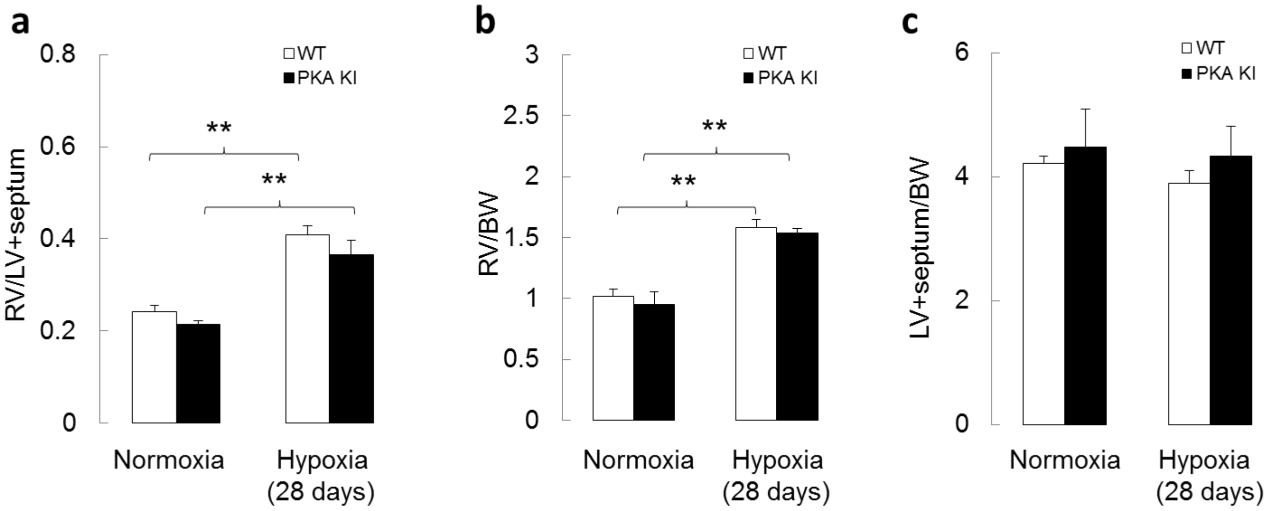

Fig. S8 Images of immunoblots before and then after any cropping or rescaling, together with molecular weight markers, for each protein documented in Figure 1a-b. In some cases, the aspect ratio of the original immunoblots was altered to enable a concise multi-panel figure with a consistent presentation style.

Fig. 1a Disulfide-PKGIα

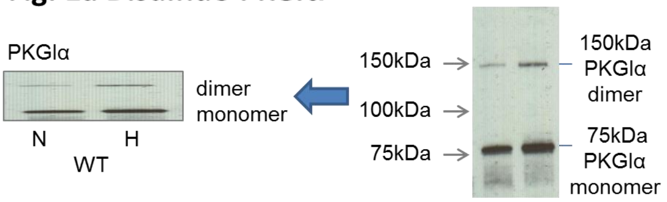

Fig. 1a PKGIα

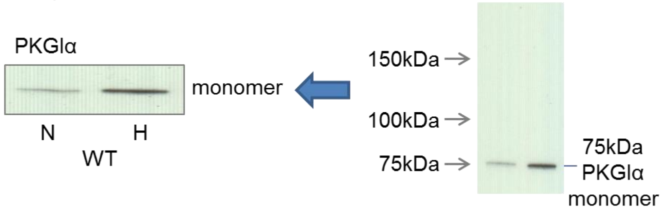

Fig. 1a CSE

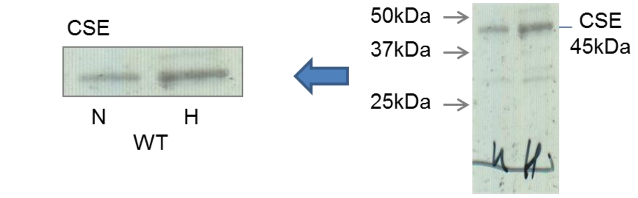

Fig. 1a Nox4

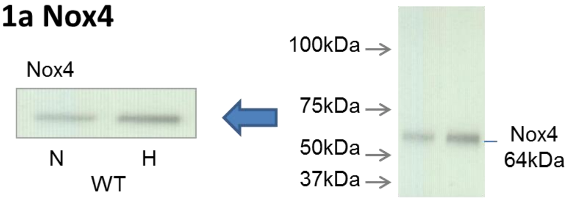

Fig. 1a SOD3

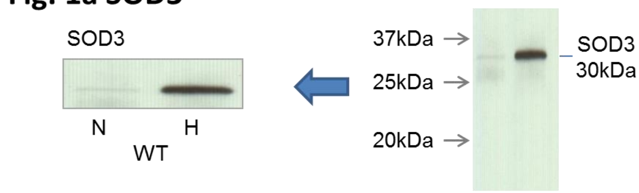

Fig. 1a Tubulin

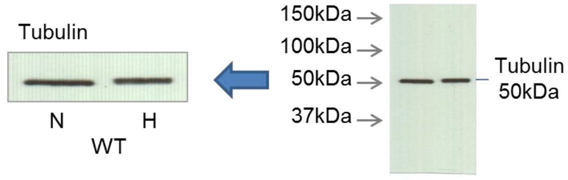

Fig. 1b Disulfide-PKGIα

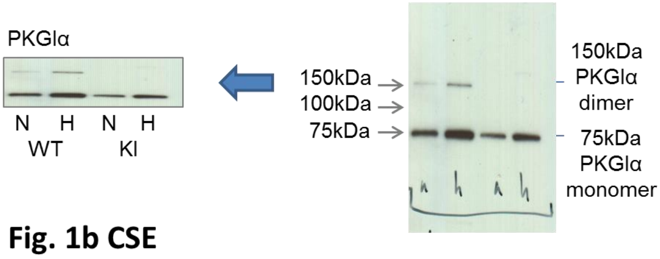

Fig. 1b PKGIα

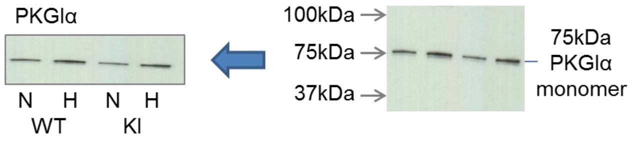

Fig. 1b CSE

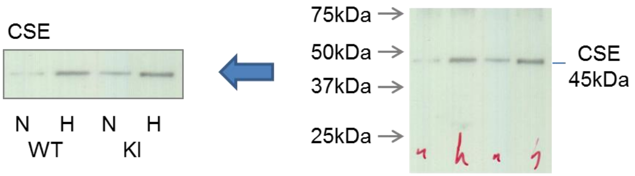

Fig. 1b Nox4

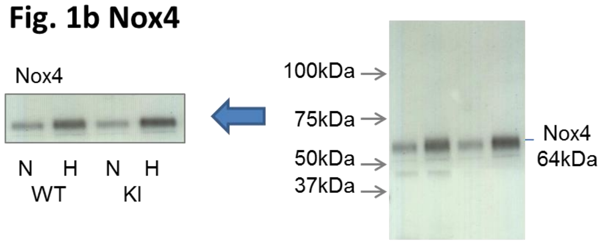

Fig. 1b SOD3

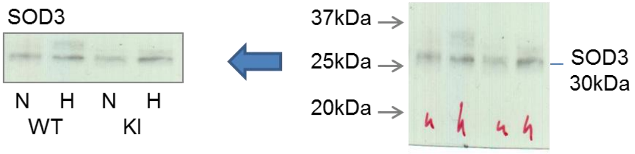

Fig. 1b Tubulin

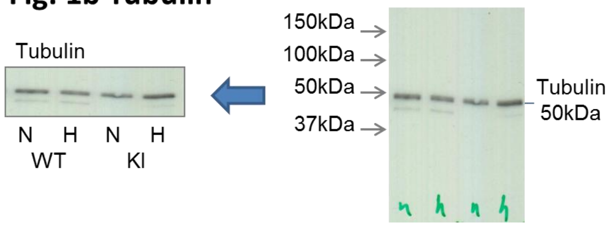

Fig. S9 Images of immunoblots before and then after any cropping or rescaling, together with molecular weight markers, for each protein documented in Figure 1c-d. In some cases, the aspect ratio of the original immunoblots was altered to enable a concise multi-panel figure with a consistent presentation style.

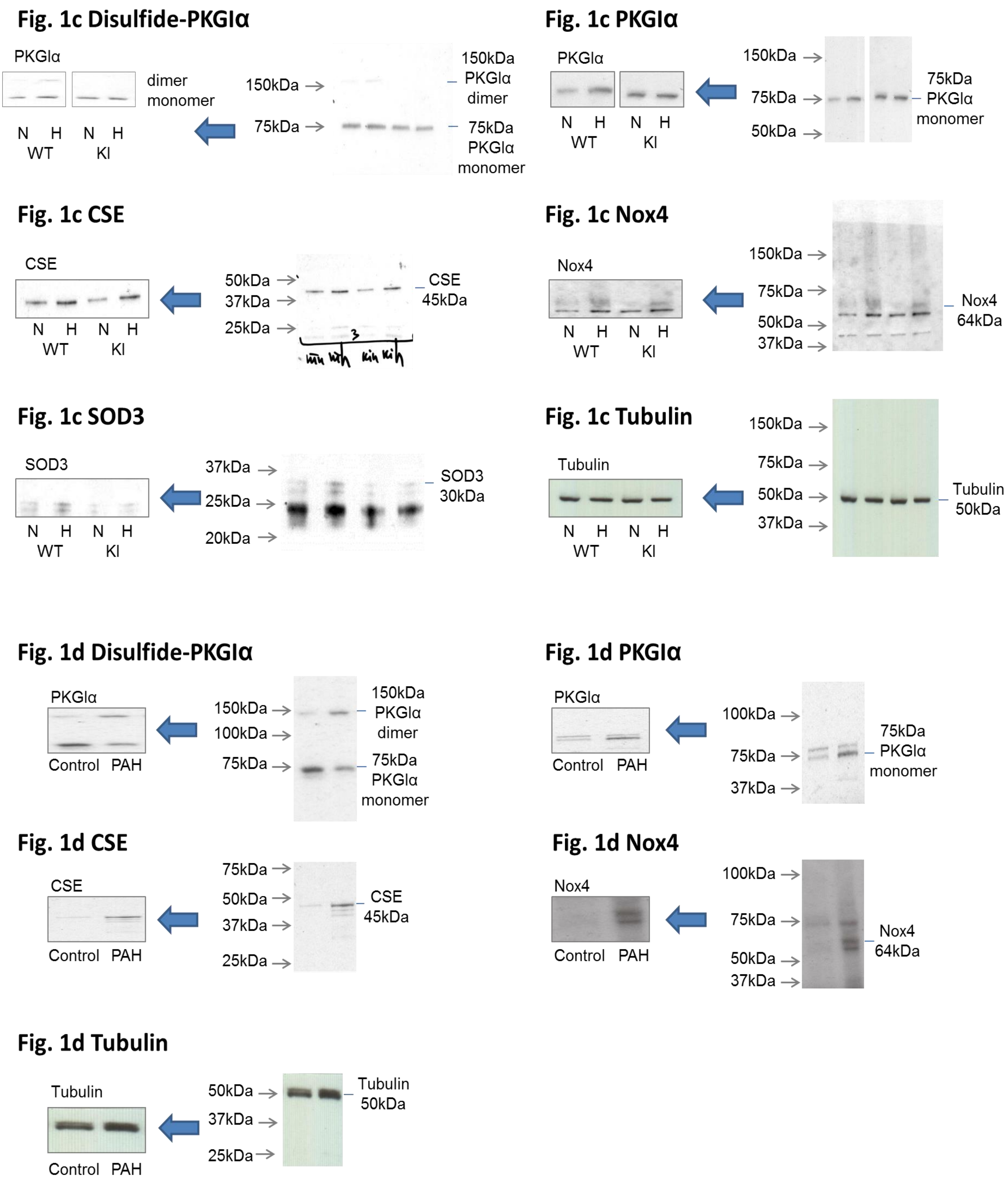

Fig. S10 Images of immunoblots before and then after any cropping or rescaling, together with molecular weight markers, for each protein documented in Figures 2-4. In some cases, the aspect ratio of the original immunoblots was altered to enable a concise multi-panel figure with a consistent presentation style.

Fig. 2a Disulfide-PKGIα

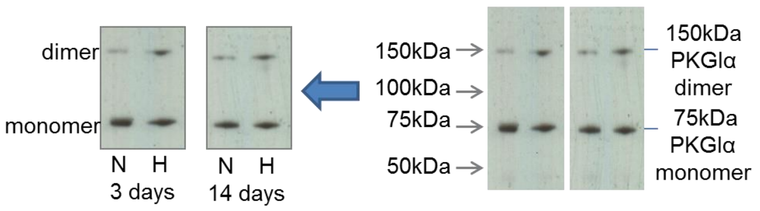

Fig. 3a Disulfide-PKGIα

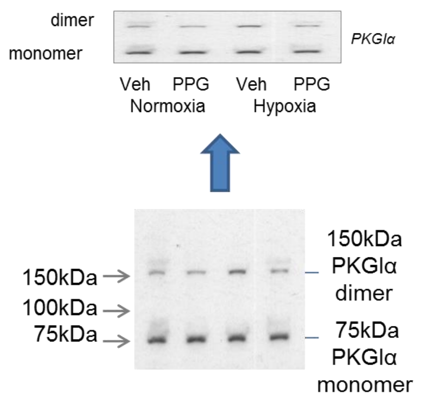

Fig. 3b CSE

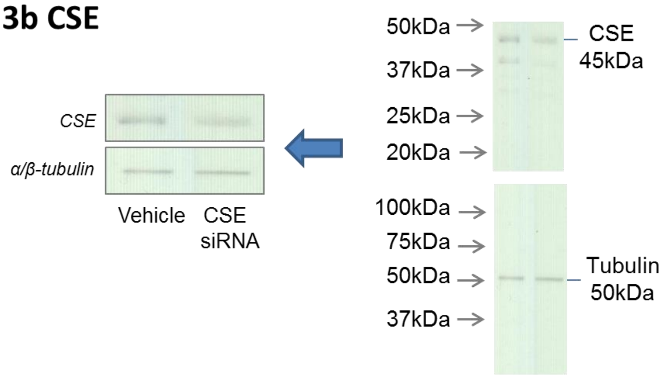

Fig. 3b PKGIα

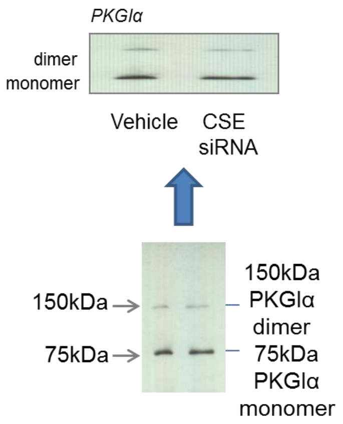

Fig. 3c PKGIα

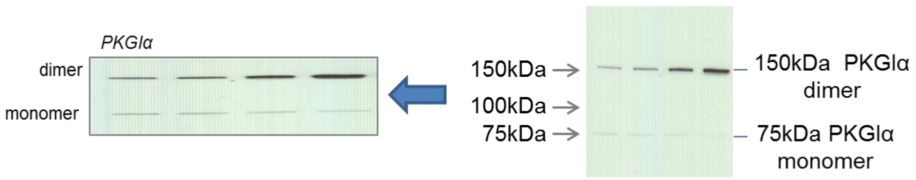

Fig. 4d pVASP and pMLC

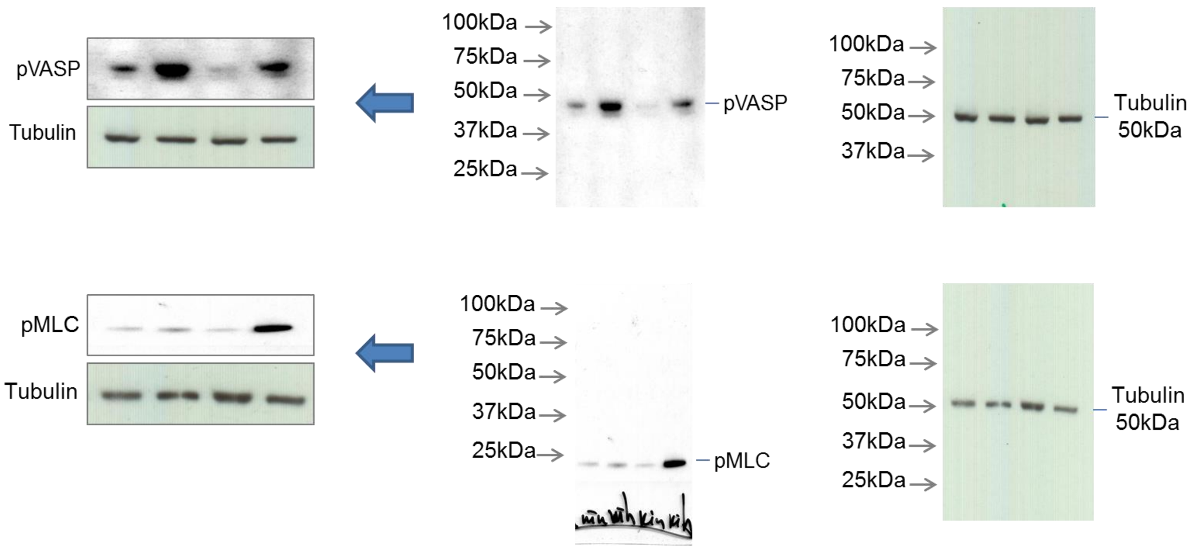

Fig. S11 Images of immunoblots before and then after any cropping or rescaling, together with molecular weight markers, for each protein documented in Figure 5. In some cases, the aspect ratio of the original immunoblots was altered to enable a concise multi-panel figure with a consistent presentation style.

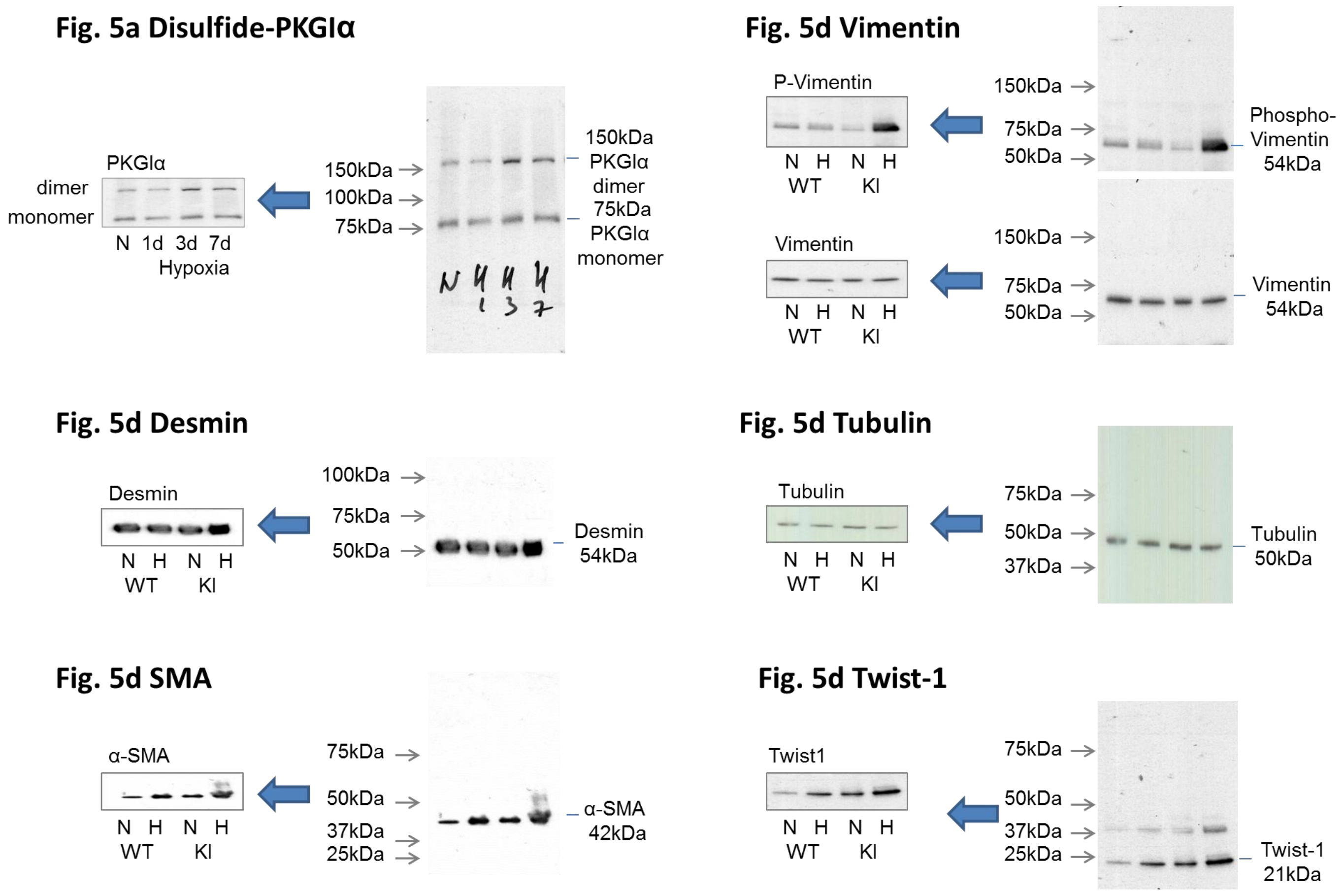

SI Materials and Methods

Animals, induction of hypoxic pulmonary hypertension and treatment

All animal procedures were performed in accordance with the Home Office Guidance on the Operation of the Animals (Scientific Procedures) Act 1986 in the United Kingdom and were approved by the King's College Animal Welfare and Ethical Review Body. Mice constitutively expressing PKGIα Cys42Ser were produced on a pure C57BL/6 background by Taconic Artemis as described (1, 2) and bred on-site. Age- and body weight–matched WT or PKGIα Cys42Ser KI male offspring were used in most of the studies. In some experiments, age and body-weight matched adult C57BL/6 male mice were purchased from Charles River (UK), as highlighted in more details in the Results section. Animals had ad libitum access to standard chow and water and were kept in a specific pathogen-free conditions under a 12-hour day/night cycle at 20°C and 60% humidity prior to hypoxic exposure. Hypoxic pulmonary hypertension was induced by exposing mice to normobaric hypoxia (10% of inspired O_2) in a large ventilated chamber (Biospherix, Ltd) **(Fig. S1).** The CO_2 level was monitored continuously with CO_2 meter and soda-lime. Fresh cage, water and food change were performed once every 7-10 days for all the animals.

In some experiments mice were implanted subcutaneously with osmotic mini-pumps (2002, Alzet®, Charles River, UK) for continuous drug delivery during 14 days. Pumps were primed overnight at 37°C prior to implantation prior to surgery which was performed in mice anesthetised with 2% isoflurane (Centaur Services) in 0.5 l of oxygen per minute with peri-operative analgesia (methadone, 1 mg/kg of body mass, Comfortan, Dechra, UK). Chemicals for osmotic mini-pump experiments, such as L-PPG, K_2S_x and NaHS were obtained from Sigma (UK). Silencer Select Pre-Designed In Vivo Ready siRNA (Assay ID s99002) was obtained from Thermo Fisher Scientific, Life Technologies and delivered in vivo with a single tail vein injection in a final concentration 1.3 mg/kg by using Invivofectamine Lung (Life Technologies, Thermo Fisher Scientific), according to the manufacturer instructions.

Echocardiography and hemodynamic measurements

Cardiac function and pulmonary arterial stiffness was assessed by non-invasive cardio-vascular-pulmonary imaging. Mice were anesthetised with 2% isoflurane in 0.5 l of oxygen per minute and examined using a non-invasive high resolution Vevo 770 echocardiography system (RMV707B, VisualSonics, Toronto, ON, Canada) with a RMV-707B transducer running at 30 MHz. The core body temperature was maintained at 37°C with a feedback-regulated body temperature probe. High resolution, two-dimensional B-mode and M-mode images were obtained at the level of the papillary muscles and further analyzed offline with Vevo Software (VisualSonics) as before (2). Doppler probe and B-mode images were employed to assess pulmonary arterial flow parameters as described (3) distally of pulmonary trunk bifurcation. Upon completion of echocardiographic assessment, animals while still under general anaesthesia were placed on a complementary surgical table and intra-tracheal ventilation was initiated. The chest was opened, 1.2F pressure catheter (Scisence Inc) was placed into the right ventricle (RV) and the RV systolic pressure was recorded for 20 min after stabilisation at the core body temperature 37°C. In some experiments, after completion of echocardiography imaging, mice were euthanized, and blood was rapidly sampled from the inferior vena cava and immediately analysed for hematocrit and hemoglobin levels using an iSTAT blood biochemistry analyser with EC8+ iSTAT cartridges (Abaxis, UK).

Assessment of right ventricular hypertrophy

The RV free wall was separated from the left ventricular (LV) wall and ventricular septum. RV hypertrophy was expressed as the ratio of RV free wall weight to the free LV wall plus ventricular septum (LV+S) weight. Left lung lobe was fixed for histological assessment in 4% formalin at 10 cm of H_2O. At the same time right lung was snap-frozen in liquid nitrogen for further biochemical analysis. In some experiments the whole hearts were fixed in 4% formalin for histological assessment of RV hypertrophy.

Cell studies

Human PASMCs were purchased from ScienCell Research Laboratories (Carlsbad, USA). For CSE siRNA validation experiments, mouse PASMC were isolated from C57BL/6 mice as described (4). Cells were cultured in Dulbecco's Modified Eagle Medium (DMEM) supplemented with 10% foetal bovine serum, penicillin and streptomycin. All experiments were performed using human PASMCs of passages 5-8, or mouse PASMCS of passages 2-3 which were grown on 12-well plates in an incubator at 37°C with a 95% O_2:5% CO_2 environment. Once confluent, human PASMCs were treated with or without potassium polysulfides (K_2S_x) in a concentration range 0.1-1 ng/ml for 15 min. Mouse PASMC were transfected with silencer select pre-designed CSE siRNA (Assay ID s99002, Thermo Fisher Scientific, Life Technologies) in a concentration range 2-100 nmol/l for 48 hours by using DharmaFECT 2 Transfection Reagent (Dharmacon, USA). Immunoblots were probed with primary antibodies to PKGI (1:1,000, Enzo Life Sciences), CSE (1:1,000, Novus Biologicals) or GAPDH-HRP (1:1000, Cell Signalling Technologies).

Wire tension myography

Age- and body weight–matched WT or PKGIα Cys42Ser KI male mice were injected intraperitoneally with 45 mg/kg pentobarbital mixed with heparin (1000 IU/kg). The heart and lung block was removed and second order pulmonary arteries were dissected and immersed in cold (4 °C) Krebs-Ringer solution (2.5 mM $CaCl_2 \cdot 2H_20$, 118 mM NaCl, 11 mM glucose D(+), 25 mM $NaHCO_3$, 4.7 mM KCl, 1.18 mM KH_2PO_4, 1.16 mM $MgSO_4$). Vessels were cleaned, cut into 3-mm-long vessel rings and mounted in a Danish Myo Technology wire tension myograph. Rings were bathed in Krebs-Ringer solution at 37 °C and supplied with a continuous gas mixture of 95% O_2 and 5% CO_2. Before treatment vessels were stretched to the optimal pre-tension condition using the Danish Myo Technology normalization module. Rings were incubated for 30 min in Krebs before the addition of U-46619 (0.1µM) to cause vasoconstriction, followed by a cumulative dose-dependent relaxation to H_2O_2 up to 100 µM. Results were analysed using LabChart software.

Isolated perfused lung

Age- and body weight–matched WT or PKGIα Cys42Ser KI male mice were injected intraperitoneally with 20 mg/kg pentobarbital mixed with heparin (1000 IU/kg). The lungs of anesthetised mice were ventilated with air in situ (IPL-1, Harvard Apparatus, USA) at a constant end-expiratory pressure (12 to 15 cm H_2O) and perfused in situ in the open chest with RPMI 1640 medium (containing 4% hydroxyethyl starch (HES 200) and Ultraglutamine, no phenol red) at a flow rate of 2 mL/min with a non-pulsatile pump (Ismatec REGLO), as described previously (5, 6). After 20 minutes, each preparation was challenged with thromboxane A2 receptor agonist U-46619 by adding it to the perfusate. This resulted in a

stable pulmonary vasoconstriction and was followed by a dose-dependent relaxation to H_2O_2 up to 100 μM. Results were analysed using LabChart software.

Tissue preparation, biochemical, metabolic and genetic analyses

Tissues for molecular analysis were harvested in complementary gloved hypoxic chamber (Biospherix, Ltd) under oxygen concentration that mice were exposed to (10% or room air). Western immunoblotting was carried out after protein separation by SDS–PAGE, with the addition of 100 mM maleimide in lysis buffer to alkylate thiols preventing disulfide exchange. Immunoblots were probed with primary antibodies to PKGI (1:1,000, Enzo Life Sciences), CSE (1:1,000, Novus Biologicals), phospho-VASP (1:1,000, Ser239, Cell Signalling), phospho-myosin light chain 2 (1:1,000, Ser19, Cell Signalling), SOD1, SOD2 and SOD3 (1:1000, Cell signalling), Nox4 (1:500, custom-made antibody provided by Prof Ajay Shah), desmin (1:1000, Cell signalling), α-SMA (1:1000, Dako UK Ltd), phospho (1:1,000, Ser55, Abcam Ltd) and total vimentin (1:1,000, Abcam Ltd), Twist (1:1,000, Santa Cruz) and DHFR (1:1,000, Abcam Ltd). Horseradish peroxidase-linked secondary antibody (1:1,000, Cell Signalling) and enhanced chemiluminescence (ECL) reagent (Pierce) were used. The dimensions of some immunoblots were slightly altered digitally, resulting in a change in their aspect ratio. This was done to achieve a consistent format for each immunoblot, aiding in the generation of a concise multi-panel figure. Unaltered, original digitized immunoblots were quantitatively analysed using Gel-Pro Analyser 3.1. Images of immunoblots before and then after any cropping or rescaling, together with molecular weight markers, for each protein are showed in **Figures S8-S11.** PKGIα disulfide dimer level was determined by measuring the amount of this protein in the reduced monomeric and disulfide oxidized dimeric states and calculating the percentage oxidized, as described previously (2, 7).

Analysis of a snapshot steady-state reactive oxygen and sulfur species was performed in lung and plasma tissues of mice subjected to chronic hypoxia for 3 and 14 days. H2O2 amount was assesses by Amplex Red hydrogen peroxide assay kit (Invitrogen, Carlsbad, USA), according to the manufacturer instructions.

The low-molecular-weight sulfur and persulfide species were analysed as described earlier (60). The thiol and hydropersulfide-containing compounds in mouse tissues were alkylated with β-hydroxyl-ethyl-iodoacetamide (HPE-IAM) and stabilized as HPE-IAM adducts. Briefly, the lung and plasma were homogenized in a cold methanol solution containing 5 mM HPE-IAM, after which lysates were incubated at 37°C for 20 min. After centrifugation, aliquots of the supernatants of the lysates were diluted with 0.1% formic acid containing known amounts of isotope-labelled internal standards, which were then analysed via LC-ESI-MS/MS for persulfide determination. A triple quadrupole (Q) mass spectrometer LCMS-8050 (Shimadzu) coupled to the Nexera UHPLC system (Shimadzu) was used to perform LC-ESI-MS/MS. Various persulfide HPE-IAM adducts and oxidized forms of persulfides such as GSSSG were identified and quantified by means of the multiple reaction monitoring according to our previous report (60). The protein persulfidation was also identified as reported earlier (60). The mouse lung and plasma were homogenized with a Polytron homogenizer with RIPA buffer containing 5 mM HPE-IAM, followed by centrifugation (14,000 × g, 10 min, 4 °C). The supernatants were applied to a PD SpinTrap G-25 column equilibrated with RIPA buffer to remove low molecular weight compounds, after which protein (0.4 mg/ml) was digested with 3 mg/ml pronase in the presence of 1 mM HPE-IAM for 1 h. After addition of 0.1% formic acid to

the pronase digest and centrifugation, the supernatants were subjected to LC-ESI-MS/MS described above.

Affymetrix analysis was performed as before (8). Briefly, lungs from age- and body weight–matched WT or PKGIα Cys42Ser KI male mice subjected to normobaric hypoxia for 3 days, and kept in liquid nitrogen prior to analysis. After 3 days, mice were injected intraperitoneally with 45 mg/kg pentobarbital mixed with heparin (1000 IU/kg), and left lung lobes were dissected, washed in saline, rapidly frozen in liquid nitrogen until analysis. mRNA was extracted using a RNeasy Plus Universal Kit (Qiagen). About 50 ng of total RNA was processed using an Ovation WTA PicoSL V2 kit (NuGEN). About 5 µg of SPIA-amplified complementary DNA was fragmented and biotin-labelled using the Encore Biotin module (NuGEN), and hybridization cocktails prepared as recommended by NuGEN. Samples were hybridized to Mouse Gene 2.0 ST arrays using standard protocols as recommended by the manufacturer (Affymetrix). Arrays were scanned using GCS3000 scanner and the resulting image intensity (CEL) files were processed using the RMA-sketch algorithm in Expression Console software (Affymetrix), to generate normalized, background-corrected, summarized data files (CHP format). All chips passed basic data quality control checks as suggested by Affymetrix. Pathway analysis was performed independently by the core genomics facility under the guidance of Dr Matthew Arno (Genomics Centre Manager) using pathway analysis (Thomson Reuters systems biology solutions).

Tissue preparation and immunohistochemistry

Mouse tissues were fixed in 4% formalin/PBS, embedded in paraffin (FFPE), sections were cut at 2 µm and stained with hematoxylin and eosin according to standard procedures. For immunohistochemical staining the Ventana Benchmark XT machine (Ventana, Tuscon, Arizona, USA) was used. Briefly, deparaffinised sections were boiled for 30 to 60 min in 10 mM citrate buffer, pH 6.0, for antigen retrieval. Primary antibody against α-SMA (α-smooth muscle actin, Dako Clone 1A4, #M0851; 1:100) were diluted in 5 % goat serum (Dianova Immundiagnostic, Hamburg, Germany), 45 % Tris buffered saline pH 7.6 (TBS) and 0.1 % Triton X-100 in antibody diluent solution (Zytomed, Berlin, Germany). Sections were then incubated with primary antibody for 1 hr. Anti-mouse histofine Simple Stain MAX PO Universal immunoperoxidase polymer (Nichirei Biosciences, Wedel, Germany) were used as secondary antibodies. Detection of secondary antibodies and counter staining was performed with an ultraview universal 3,3´-Diaminobenzidine (DAB) detection kit from Ventana (Ventana, Tuscon, Arizona, USA) according to the standard settings of the machine. Tissues were counterstained with hematoxylin. Slides were counterstained with 4',6'-diamidino-2- phenylindole (DAPI). Representative images were taken with a Leica DMD108 digital microscope.

For immunofluorescence analysis, mounted sections (2 µm) were dewaxed and antigen retrieval was performed for 30 min at 96°C in 10mM citrate buffer pH6.0. Sections were washed once, permeabilized with 0.2 % TritonX 100 (Roche), washed again, and blocked in blocking buffer (Protein-Free T20 (TBS) Blocking buffer #37071, Thermo Fischer) for 1 h. Anti-CD31 antibody (Dianova Clone SZ31; 1:50) and anti-α-SMA (see above) were incubated at 4°C overnight. Afterward, sections were intensively washed followed by incubation with Alexa488-conjugated secondary anti-rat and Alexa647-conjugated secondary anti-mouse antibody for 1.5 hours at room temperature, respectively. After repeated washing, sections were mounted with DAPI-Fluoromount-G (SouthernBiotech, Birmingham, USA). Data

acquisition was performed using a Leica Sp5 confocal microscope and Leica application suite software (LAS-AF-lite).

Human PAH samples

Peripheral lung tissue samples were obtained from explanted lungs of eight patients with pulmonary arterial hypertension (mean age ± SD: 39.4 ± 12.4 years; 4 females, 4 males). Cardiac measurements were obtained by right heart catheterization. Mean pulmonary arterial pressure of these patients was 58 ± 7.7 mmHg; mean systolic arterial pressure 95 ± 12 mmHg; and 6 minute walking distance 400 ± 50 m. Six non-utilized donor lungs or lobes fulfilling transplantation criteria served as controls (mean age ± SD: 56.0 ± 12.9 years; 4 females, 2 males). All lung tissue samples were collected in frame of the European IPF registry (eurIPFreg) and provided by the UGMLC Giessen Biobank, member of the DZL Platform Biobanking. Informed consent was obtained in written form from each subject. The study protocol was approved by the Ethics Committee of the Justus-Liebig-University School of Medicine (No. 111/08 and 58/15). All PAH diagnoses were made according to the WHO guidelines.

Statistics

Statistical analysis included 2-way ANOVA and non-paired t-tests. Results are presented as mean±SEM. Sample size required to achieve statistical significance was estimated using a power calculation based on past experience of anticipated differences between groups and group variance. Data from in vitro studies were only excluded if there was a technical failure. Differences between groups, in studies that were not blinded or randomized, were assessed using analysis of variance followed by a t-test. In addition, where more than two groups were compared, they were assessed using a Bonferroni correction. Differences were considered significant at the 95% confidence level.

References

1. Prysyazhna O, Rudyk O, & Eaton P (2012) Single atom substitution in mouse protein kinase G eliminates oxidant sensing to cause hypertension. Nat Med 18(2):286-290.

2. Rudyk O, et al. (2013) Protein kinase G oxidation is a major cause of injury during sepsis. Proc Natl Acad Sci U S A 110(24):9909-9913.

3. Thibault HB, et al. (2010) Noninvasive Assessment of Murine Pulmonary Arterial Pressure Validation and Application to Models of Pulmonary Hypertension. Circ-Cardiovasc Imag 3(2):157-163.

4. Lee KJ, Czech L, Waypa GB, & Farrow KN (2013) Isolation of pulmonary artery smooth muscle cells from neonatal mice. J Vis Exp (80):e50889.

5. Zhao L, et al. (2001) Sildenafil inhibits hypoxia-induced pulmonary hypertension. Circulation 104(4):424-428.

6. Krabbe J, et al. (2018) The effects of hydroxyethyl starch and gelatine on pulmonary cytokine production and oedema formation. Sci Rep 8(1):5123.

7. Rudyk O, Prysyazhna O, Burgoyne JR, & Eaton P (2012) Nitroglycerin fails to lower blood pressure in redox-dead Cys42Ser PKG1alpha knock-in mouse. Circulation 126(3):287-295.

8. Burgoyne JR, et al. (2015) Deficient angiogenesis in redox-dead Cys17Ser PKARI alpha knock-in mice. Nature Communications 6.
